# Effects of macro vs. micro initialization and ocean initial-condition memory on the evolution of ensemble spread in the CESM2 large ensemble

**DOI:** 10.1007/s00382-024-07553-z

**Published:** 2024-12-24

**Authors:** Clara Deser, Who M. Kim, Robert C. J. Wills, Isla R. Simpson, Steve Yeager, Gokhan Danabasoglu, Keith Rodgers, Nan Rosenbloom

**Affiliations:** 1 NSF National Center for Atmospheric Research, Boulder, CO USA; 2 Institute for Atmospheric and Climate Science, ETH Zürich, Zürich, Switzerland; 3 WPI-Advanced Institute for Marine Ecosystem Change, Tohoku University, Sendai, Japan; 4 Climate and Global Dynamics Laboratory, NCAR, 1850 Table Mesa Drive, Boulder, CO 80305 USA

## Abstract

“Single Model initial-condition Large Ensembles” (SMILEs) conducted with Earth system models have transformed our ability to quantify internal climate variability and forced climate change at local and regional scales. An important consideration in their experimental design is the choice of initialization procedure as this influences the duration of initial-condition memory, with implications for interpreting the temporal evolution of both the ensemble-mean and ensemble-spread. Here we leverage the strategic design of the 100-member Community Earth System Model version 2 (CESM2) SMILE to investigate the dependence of ensemble spread on the method of initialization (micro- vs. macro- perturbations) and the effects of ocean initial-condition memory. We find that the evolution of ensemble spread in 10-year low-pass filtered data is relatively insensitive to the method of initialization beyond the second decade, with the notable exception of the tropical Indo-Pacific in the 4th decade, when macro-initialization significantly enhances ensemble spread, possibly as a result of a state-dependent response to major volcanic activity. Initial-condition memory associated with the chosen Atlantic Meridional Overturning Circulation (AMOC) states unfolds in two stages: First, in the North Atlantic lasting 4–5 decades, and subsequently, in the Indo-Pacific sector of the Southern Ocean appearing 35-years after initialization and lasting 3–4 decades. Known AMOC dynamics explain the first stage, but the role of AMOC and the mechanisms responsible for the delayed appearance of initial-condition memory in the Southern Ocean remain to be fully elucidated. Implications and recommendations for the design of future SMILEs are provided.

**Supplementary Information:**

The online version contains supplementary material available at 10.1007/s00382-024-07553-z.

## Introduction

“Single Model Initial-condition Large Ensembles” (SMILEs) conducted with global coupled Earth system models have become an indispensable tool for the study of climate variability and change (e.g., Deser et al. 2012; Kay et al. 2015; Deser et al. 2020; Lehner et al. 2020). A hallmark of SMILEs is the ability to unambiguously separate the evolving characteristics of internal climate variability from those of the forced response at local and regional scales in a given model. This separation is achieved by conducting many simulations with the same model and the same radiative forcing protocol (historical and/or future scenario) but varying the initial conditions. The resulting ensemble spread is thus solely attributable to differences in the trajectories of internal variability across the individual members. Once the memory of the initial conditions is lost, the sequences of internal variability will be randomly phased amongst the members such that the forced component at a given location and time can be estimated by averaging the simulations together, provided a sufficient ensemble size, and the internal component in each member can be obtained as a residual from the forced component. The accuracy of the estimated forced response depends on the number of members available for averaging and may differ according to the spatio-temporal scale and quantity of interest. For example, very large ensembles (order 100 members) may be required to detect forced changes in the characteristics of internal variability (e.g., Milinski et al. 2020; Rodgers et al. 2021) while smaller ensembles (order 10 members) may be sufficient for detecting forced changes in the mean state (e.g., Deser et al. 2012; Fasullo and Nerem 2018; Wills et al. 2020). The proliferation of SMILEs in recent years attests to their enormous value for studying the evolving nature of climate variability and change at local and regional scales, and providing quantitative information needed for risk assessment and adaptation/planning purposes.

An important aspect of the experimental design of SMILEs is the choice of initialization procedure, as this influences the duration of initial-condition memory and has implications for the evolution of ensemble spread (Drotos et al. 2015 and 2017). Many SMILEs employ a so-called “micro-initialization” procedure by introducing a small random perturbation to the initial atmospheric temperatures (on the order of the model’s numerical round-off error, e.g., 10^− 14^ K; Deser et al. 2012 and Kay et al. 2015) or to the initial ocean temperatures at a single, randomly chosen grid cell (on the order of 10^− 3^ K; Hawkins et al. 2016). Other SMILEs use a so-called “macro-initialization” approach by selecting different coupled model states taken from a long pre-industrial control (Pictl) simulation. These Pictl states are typically chosen at random a decade or more apart depending on the availability of the restart files (Maher et al. 2019; Bonnet et al. 2021; Singh et al. 2023; Voldoire et al. 2019; Doscher et al. 2021; Boucher et al. 2020), although more strategic approaches based on maximizing spread in the Atlantic Meridional Overturning Circulation (AMOC) or in ocean heat content (OHC) contrasts between the Pacific and Atlantic have also been employed (see Hawkins et al. 2016 and Stevenson et al. 2023; respectively). Finally, some SMILEs employ a combination of micro- and macro-initialization procedures (Hawkins et al. 2016; Kirchmeier-Young et al. 2017; Rodgers et al. 2021; Singh et al. 2023). Regardless of the criteria used for selecting macro initial states from a Pictl simulation, long-term drift and/or decadal-to-centennial quasi-periodicities need to be considered. Indeed, long-term drift was the main source of spread in OHC of the Southern Ocean across the set of macro initial states in Singh et al. (2023). Further, Stevenson et al. (2023) caution that many models exhibit multi-centennial OHC variability in their Pictl simulations, thereby introducing long-term memory effects associated with the macro states used to initialize historical SMILE simulations. The impact of long-term drift and/or low-frequency variability in Pictl simulations is typically overlooked in analyses of SMILEs based on macro-initialization (Bonnet et al. 2021 is a notable exception).

The extent to which the method of initialization affects the behavior of ensemble spread through time in a given model has, to the best of our knowledge, been addressed in only two studies to date: Hawkins et al. (2016) and Singh et al. (2023). Hawkins et al. (2016) employed a low-resolution intermediate complexity model (5° x 7.5° with 11 vertical levels in the atmospheric component and 2.5° x 3.75° with 20 vertical levels in the ocean component) under idealized radiative forcing (1% per year compound increase in CO_2_ over 140 years) and focused on European climate. Singh et al. (2023) used a relatively high-resolution Coupled Model Intercomparison Project phase 5 (CMIP5)-era model (approximately 2° in the atmosphere and 1° in the ocean) under historical and future (RCP8.5) radiative forcing and focused on the Southern Ocean. Hawkins et al. (2016) concluded that “an ensemble using a range of different oceanic initial conditions produces a larger spread in temperature trends than ensembles using a single ocean initial condition at all lead times”, while Singh et al. (2023) found “a discernible impact of varying ocean initial conditions on ensemble variance over the Southern Ocean is evident throughout the full 150 simulation years of the ensemble”. The extent to which the conclusion of Hawkins et al. (2016) holds for more complex models and radiative forcing scenarios remains to be ascertained. Similarly, the predominant influence of model drift on the evolution of OHC from the macro initialization states used in Singh et al. (2023) calls for additional investigation into initial-condition memory effects after accounting for model drift. A recent study by Stevenson et al. (2023) provides a comparison of ensemble spread behavior in upper-ocean heat content and surface temperature across SMILEs initialized with different approaches, but model differences in addition to differences in initialization procedures prevented them from drawing definitive conclusions.

Here, we employ a recent state-of-the-art 100-member SMILE strategically designed to allow investigation of the dependence of ensemble spread on micro vs. macro initialization as well as ocean initial-condition memory effects (Rodgers et al. 2021). This SMILE was conducted with the Community Earth System Model version 2 (CESM2), a CMIP6-era model at nominal 1° resolution (Danabasoglu et al. 2020). Each member of the CESM2 SMILE starts from a different initial condition as described in Sect. 2 and is subject to the same radiative forcing protocol (historical from 1850 to 2014 and SSP3.70 from 2015 to 2100), with the exception of the biomass burning emissions in the late 20th and early 21st centuries being temporally smoothed in the second set of 50 members compared to the first 50 (Rodgers et al. 2021), although this does not impact the period of simulation that is of interest here. We focus our analysis on the evolution of upper ocean temperatures and surface climate variables over the first 6 decades of the historical simulations, when the impact of initialization is expected to be most pronounced. Although there is relatively little drift in the CESM2 Pictl, we take care to remove this drift from the historical SMILE simulations before assessing their ensemble spread and initial-condition memory. Our aim is to document the degree to which the method of initialization and choice of ocean initial conditions affect ensemble spread in the CESM2 SMILE. We hope that our findings will stimulate future investigations of the physical mechanisms governing the sensitivity of ensemble spread to these methodological choices. Finally, our results may help to inform the design of future SMILEs and provide context for analyses of internal variability and forced responses in SMILEs.

The rest of this study is organized as follows. The CESM2 SMILE experimental design is presented in Section 2a. Methods for removing drift based on the Pictl simulation, decomposing ensemble variance into contributions from macro vs. micro initialization, attributing ensemble variance to ocean initial-condition memory, and statistical significance testing based on bootstrapping the Pictl simulation are given in Section 2b. Results on the sensitivity of ensemble spread to the method of initialization are presented in Section 3a, and those on ocean initial-condition memory are presented in Section 3b. A discussion of mechanisms underlying initial-condition memory related to AMOC and the potential role of external forcing in enhancing ensemble spread due to macro-initialization are provided in Section 4a and 4b, respectively. Summary and implications follow in Sect. 5.

## Materials and methods

### CESM2 SMILE experimental design

The initialization strategy for the CESM2 SMILE was explicitly designed with two questions in mind: (1) Does the evolution of ensemble spread depend on the method of initialization (e.g., micro vs. macro perturbation)?; and (2) How long does near-surface climate retain memory of the initial ocean state?

**Fig. 1:**
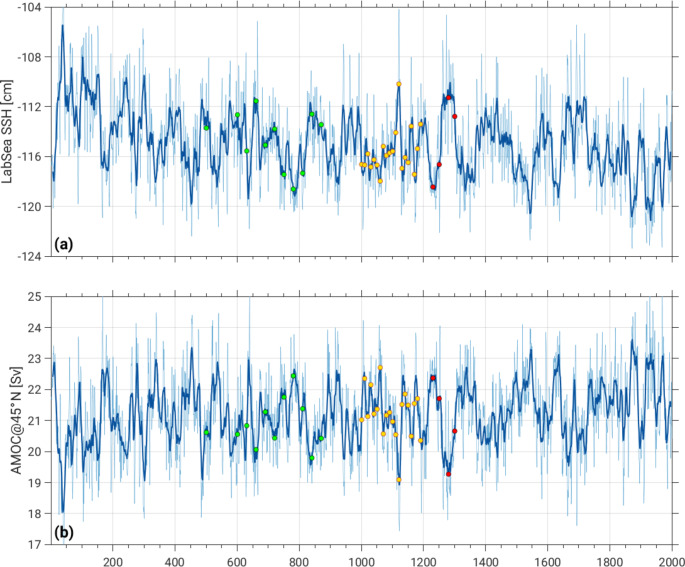
Timeseries of (**a**) LabSeaSSH and (**b**) AMOC45N from the CESM2 pre-industrial control simulation (thin curves show unsmoothed annual values and thick curves show 10-year running means). The four red circles mark the initial states used for AMOC1231, AMOC1251, AMOC1281 and AMOC1301 (see text for definitions). The 20 yellow circles mark the initial states used for Macro20, and the 11 green circles mark the initial states used for Macro11 (see text for definitions)

Four macro initial conditions for the CESM2 SMILE were selected based on contrasting states of AMOC, similar to the strategy employed in Hawkins et al. (2016), and the remaining macro initial states were randomly selected from the Pictl simulation at 10-year intervals, similar to the procedure in Singh et al. (2023). The four different AMOC initial states were selected from years 1231–1301 of the Pictl. These initial states sample minimum, maximum, and transition states of an AMOC Index representing the maximum transport at 45°N associated with the North Atlantic Deep Water (hereafter, AMOC45N) and the closely related Labrador Sea (53°-65°N, 60°-45°W) Sea Surface Height (SSH) Index (hereafter, LabSeaSSH) as shown in Fig. 1 (red circles). The rationale for choosing these indices is based on previous work which shows that low-frequency fluctuations in AMOC45N and LabSeaSSH provide a major source of decadal memory and predictability in the climate system (Yeager and Danabasoglu 2014). [Note that the selection criterion was incorrectly stated in Rodgers et al. (2021) as being based on the AMOC Index at 26°N as opposed to 45°N (their Fig. S2).] The choice of Pictl years 1231–1301 from which to select the AMOC initial states was dictated by the length of the Pictl simulation (1400 years) at the time the SMILE was configured (the Pictl was subsequently extended to 2000 years). By this time, the Pictl exhibited minimal drift in AMOC45N and LabSeaSSH, and showed a prominent quasi-centennial oscillation in both indices (Fig. 1). We aimed to sample this large-amplitude, slowly-varying fluctuation by selecting Pictl years 1231, 1251, 1281 and 1301 for the 4 AMOC initial states (indicated by the red circles in Fig. 1). Hereafter, we shall refer to these cases by their year of initialization: AMOC1231 (minimum LabSeaSSH, maximum AMOC45N); AMOC1251 (rising LabSeaSSH, declining AMOC45N); AMOC1281 (maximum LabSeaSSH, minimum AMOC45N); and AMOC1301 (declining LabSeaSSH, rising AMOC45N). Twenty-member ensembles for each AMOC initial state were generated by randomly perturbing the initial atmospheric temperatures by order 10^− 14^ K (following the “micro perturbation” protocol used for all members of the CESM1 SMILE; Kay et al. 2015). We shall refer to these 4 AMOC ensembles collectively as “AMOC4 × 20”.

The initial states for the remaining 20 ensemble members (referred to as “Macro20”) of the 100-member CESM2 SMILE were chosen from years 1001–1191 of the Pictl simulation at 10-year intervals. The LabSeaSSH and AMOC45N conditions sampled by these randomly selected Pictl years are shown in Fig. 1 (yellow circles). In addition to Macro20, 11 simulations were conducted as part of the CESM2 contribution to CMIP6 (Danabasoglu et al. 2020) prior to the design of the CESM2 SMILE. These 11 simulations, hereafter referred to as “Macro11”, were initialized from years 631–871 of the Pictl at 30-year intervals (one simulation was initialized from year 501; green circles in Fig. 1). One ensemble member was generated for each Macro20 and Macro11 initial state.

### Methods

#### De-drifting procedure based on the pre-industrial control simulation

As mentioned above, the CESM2 Pictl exhibits long-term drift, albeit much less than in many other models (see for example Singh et al. 2023). To remove this drift from each member of the CESM2 SMILE, we compute the linear trend in the Pictl simulation based on years 800–1599 and then subtract this trend from each member of the SMILE. Our de-drifting procedure is insensitive to the exact set of years used from the pictrl (not shown). We de-drift the Macro11 simulations based on the Pictl linear trend over years 500–999 (the interval from which the Macro11 initial states were drawn plus an additional ~ 120 years) to accommodate the larger drift in the early portion of the Pictl simulation compared to later years (Figs. S1 and S2); the Pictl trend is insensitive to the exact starting and ending years within this interval (not shown). An example of the raw and de-drifted timeseries of upper ocean potential temperature (T_0 − 500 m_) in the Pacific sector of the Southern Ocean (60°-78°S, 180°-70°W) is shown in Fig. S2.

#### Decomposition of ensemble variance

We investigate two complementary aspects of ensemble spread: (1) the effect of initialization method (macro vs. micro perturbation); and (2) the effect of ocean initial-condition memory in the AMOC4 × 20 ensembles. All results are based on de-drifted decadal averages or, in the case of time series, 10-year running means.

### Macro vs. micro initialization

We quantify the time-evolving ensemble spread (e.g., standard deviation across members) resulting from macro- vs. micro- initialization, hereafter denoted s_macro_(t) and s_micro_(t), respectively, as follows. We compute s_macro_(t) by combining the 20 members of Macro20 with the 11 members of Macro11 for added robustness, but similar results are found using only the Macro20 ensemble (not shown). We compute s_micro_(t) by calculating s^2^ (t) separately for each 20-member AMOC ensemble (e.g., AMOC1231, AMOC1251, AMOC1281 and AMOC1301) and then take the square-root of the average of the four s ^2^(t) values. In this way, s_micro_(t) is based on a total of 80 samples at each time step, enhancing robustness. We have verified that in each decade, the four s^2^ (t) values are statistically indistinguishable from one another at all but a few isolated locations, justifying our averaging procedure. Specifically, we compared the standard deviation across the four s^2^ (t) values to the 2.5th – 97.5th range of standard deviations determined from a bootstrapped distribution for each decade and grid box separately. This bootstrapped distribution was built from random sampling, with replacement, four 20-member sets drawn from the full 80-member pool (where the mean of each AMOC ensemble has been subtracted from each of its constituent members), calculating the variance within each 20-member set, computing the standard deviation from the square root of the average variance across the four sets, and then repeating the entire procedure 2000 times. A different bootstrapping procedure is used to assess whether s_macro_(t) and s_micro_(t) differ significantly from each other and from the Pictl (see Section 3a).

### Ocean initial-condition memory

The fraction of ensemble variance attributable to ocean initial-condition memory in AMOC4 × 20 is assessed following the approach of Singh et al. (2023). Note that these are the only ensembles for which we have multiple members (created with the micro-initialization procedure) for a given ocean initial state. First, we compute the 20-member ensemble mean [X(t)] of each AMOC ensemble (e.g., AMOC1231, AMOC1251, AMOC1281 and AMOC1301) at each time and grid box to estimate the influence of ocean initial-condition memory in each AMOC ensemble. We then compute the variance [s^2^_ocean_(t)] across the four AMOC ensemble-means [X(t)_AMOC1231_, X(t)_AMOC1251_, X(t)_AMOC1281_, and X(t)_AMOC1301_] at each time and grid box to estimate the ensemble spread arising from ocean memory. Finally, we assess the fraction of the total variance across all 80 members that is attributable to ocean initial-condition memory, denoted χ_ocean_(t) and defined as:

χ_ocean_(t) = s^2^_ocean_(t)/ s^2^_total_(t)    (1)

where s^2^_total_(t) is the total variance (due to random noise plus ocean memory) across the 80 members. We assess the statistical significance of χ_ocean_(t) against a null hypothesis of sampling fluctuations using bootstrapping of the Pictl as described in Section 3b.

## Results

### Effect of macro vs. micro initialization on ensemble spread

In this section, we survey the effects of macro vs. micro initialization on the evolution of ensemble spread in a selection of oceanic and surface climate variables over the first 6 decades (1850–1909) of the historical simulations. Specifically, we examine ensemble spread in decadal means of upper-ocean (0–500 m) potential temperature (T_0 − 500 m_), surface temperature (TS), sea level pressure (SLP) and precipitation (PR). We also compare the ensemble spread in the 31 macro-initialized historical simulations [s_macro_(t)] with internal variability diagnosed from the Pictl simulation to assess the influence of external radiative forcing on s_macro_(t). To do this, we randomly sample with replacement 31 decadal averages (mimicking the sample size for the macro simulations) from years 500–1599 of the de-drifted Pictl (recall Section 2a for the de-drifting procedure), compute their variance, and then repeat this procedure 3000 times to obtain the 2.5th – 97.5th percentile range of standard deviations of internal variability that could occur due to sampling fluctuations alone (e.g., without changes in radiative forcing). Results for T_0 − 500 m_ are described first, followed by those for TS, SLP and PR.

**Fig. 2:**
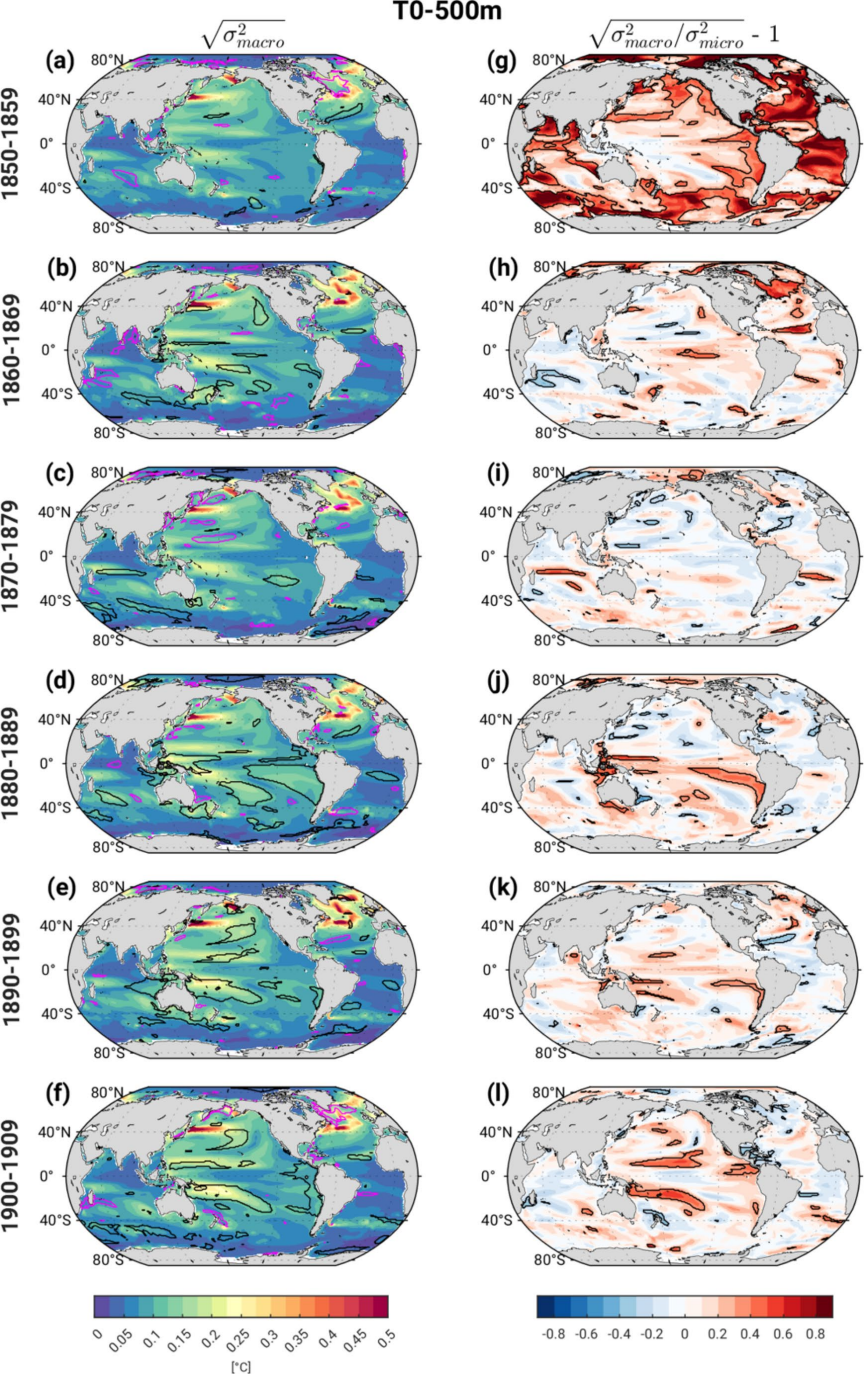
Ensemble spread in decadal-average T_0 − 500 m_ (K) over the first 6 decades of the historical simulations based on (left) macro-initialization [√s^2^_macro_(t)] and (right) compared to micro-initialization expressed as the fraction [√(s^2^_macro_(t)/s^2^_micro_(t)) − 1]. See text for definitions. The black (magenta) contours in the left column enclose regions where s_macro_(t) lies above (below) the 97.5th (2.5th ) percentile of the Pictl spread. The black contours in the right column enclose regions where the differences between s_macro_(t) and s_micro_(t) are statistically significant at the 5% confidence level based on bootstrapping the Pictl simulation

In each decade, s_macro_ T_0 − 500 m_ exhibits a zonally banded structure of westward-intensified maxima in the Pacific and Atlantic basins, consistent with well-known signatures of oceanic Rossby waves and Sverdrup balance; areas of largest spread occur along the Kuroshio Extension and Gulf Stream/North Atlantic Current regions (color shading in Fig. 2a-f). This pattern amplifies from the 1850–1860 s (Fig. 2a, b), and again from the 1880–1890 s (Fig. 2d, e). During the 1880s, a distinct secondary maximum appears over the eastern and southeastern tropical Pacific (Fig. 2d). Contours in Fig. 2a-f enclose regions where s_macro_ lies outside the 2.5th -97.5th percentile range from the bootstrapped Pictl distribution (black for > 97.5th % and magenta for < 2.5th %). In the first 3 decades, s_macro_ generally lies within the Pictl range in most areas, with some exceptions, most notably in the first decade over the western subpolar North Atlantic (SPNA) and parts of the Arctic where it falls significantly below the 2.5th % of the Pictl (magenta contours in Fig. 2a). This suggests that in this region and decade the 31 macro initial states do not span the full range of T_0 − 500 m_ variance sampled in the long Pictl simulation (a similar feature is found in the 6th decade, 1900–1909; Fig. 2f). In contrast to earlier decades, the 4th -6th decades show large-scale coherent areas of s_macro_ in the Pacific sector that exceed the 97.5th percentile of the Pictl distribution, indicating that external radiative forcing has likely enhanced ensemble spread in these regions (black contours in Fig. 2d-f). Below, we speculate that the sequential tropical Indo-Pacific volcanic eruptions of Krakatoa and Tarawera in the 1880s may have played a role in enhancing ensemble spread.

Next, we compare the impact of macro vs. micro initialization on the evolution of ensemble spread, expressed as the fractional change in standard deviation: [s_frac_ = sqrt(s^2^_macro_/s^2^_micro_) – 1]. To evaluate whether the fractional changes are statistically significant, we randomly sample with replacement 31 and 80 decadal averages from years 500–1599 of the de-drifted Pictl simulation (mimicking the sample sizes for s_macro_ and s_micro_, respectively), compute their variance (s^2^_31_ and s^2^_80_, respectively) and fractional differences [s_fracPictl_ = sqrt(s^2^_31_/s^2^_80_) – 1], and then repeat the entire procedure 3000 times to obtain the 2.5th – 97.5th percentile range of s_fracPictl_ .

Figure 2g-l shows s_frac_ T_0 − 500 m_ for each of the first 6 decades; values that are statistically significant relative to the Pictl (s_fracPictl_) are outlined in black contours. In the first decade, macro-initialization produces significantly greater spread compared to micro-initialization over much of the Arctic, Atlantic and Southern Oceans as well as over portions of the tropical Indian and eastern Pacific basins, with normalized differences on the order of 30–80% (Fig. 2g). This signal is expected due to the persistence of macro state variance (in particular, ocean initial condition memory as will be discussed below). By the second decade, significant differences are mainly limited to the SPNA and Arctic, and these diminish almost entirely by the 3rd decade (Fig. 2h-i). Surprisingly, the 4th decade shows a coherent region of statistically significant differences in ensemble spread over the eastern tropical Pacific emanating from the coast of Chile, as well as in two narrow bands on either side of the equator in the western Pacific, with values ~ 30–60% (Fig. 2j); these features decay in the 5th decade (Fig. 2k). Statistically significant differences in ensemble spread reappear in the 6th decade over the western half of the tropical Pacific, with two prominent bands of values ~ 30–60% along ~ 10°N and ~ 10°S, corresponding to locations of maximum Rossby wave activity (Capotondi et al. 2003). The areas of significant differences in macro vs. micro ensemble spreads over the tropical Pacific in the 4th -6th decades correspond roughly to the regions where external forcing has significantly enhanced macro ensemble spread (recall contours in Fig. 2d-f).

**Fig. 3:**
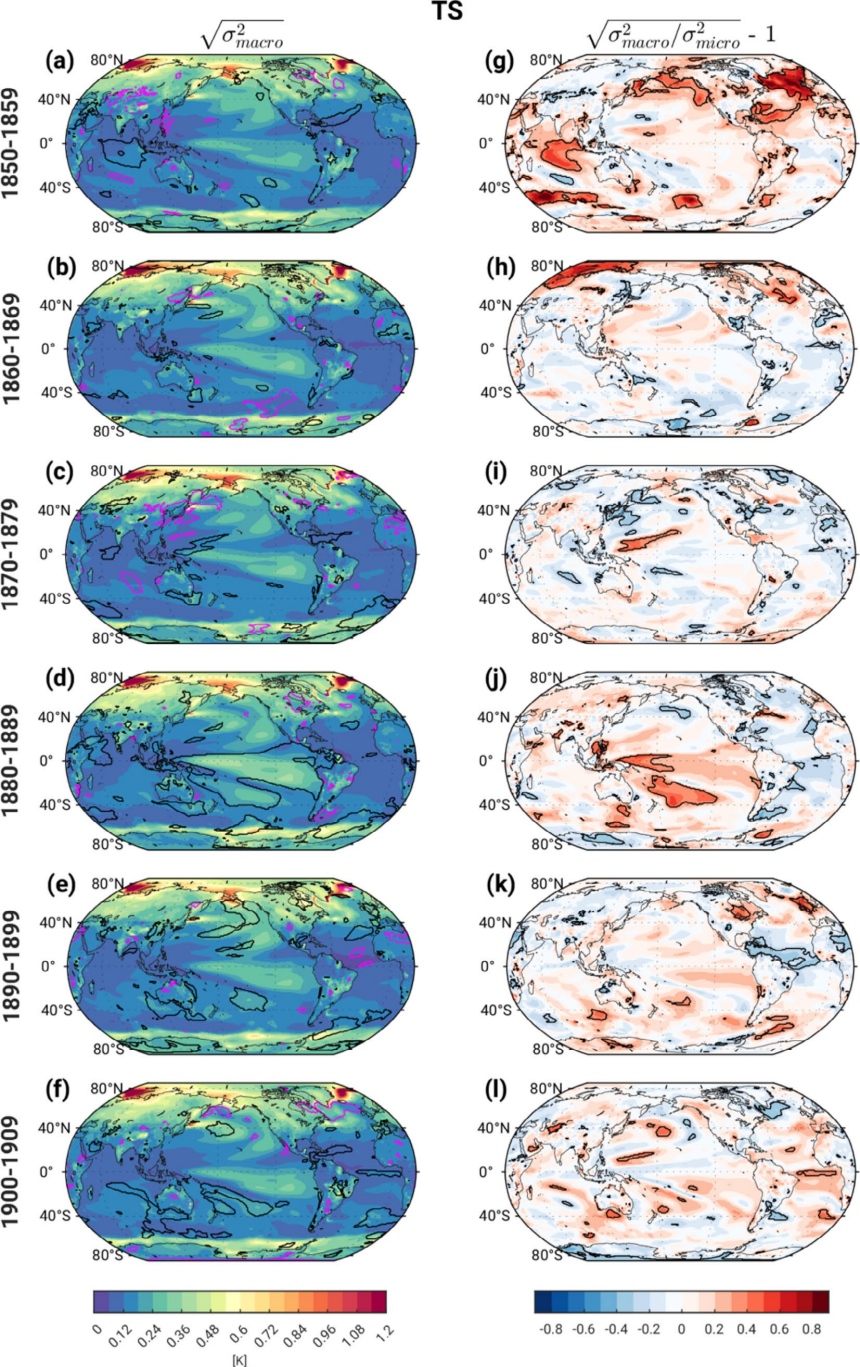
As in Fig. 2 but for surface temperature (TS)

Turning to TS, the pattern of s_macro_ features maximum values along the sea ice margins in the Arctic and Antarctic, secondary maxima over the SPNA and the northern and tropical Pacific, and a weaker enhancement over Southern Hemisphere midlatitudes especially the Pacific sector (Fig. 3a-f). The regional patterns of enhanced ensemble spread in the Atlantic and Pacific correspond to well-known modes of decadal variability, in particular Atlantic Multi-decadal Variability (AMV; Zhang et al. 2019) and Pacific Decadal Variability (PDV; Mantua et al. 1997), which CESM2 (among other models) is able to simulate (see Deser and Phillips 2023 and Capotondi et al. 2020; respectively). The amplified ensemble spread over the marginal sea ice zones reflects the high TS variability associated with transitions between sea ice and open water conditions (e.g., Deser et al. 2010). Over land, TS ensemble spread is generally largest over the northern continents as expected due to pronounced atmospheric circulation variability and associated thermal advection in middle latitudes (e.g., Deser et al. 2012; Shepherd, 2014) in addition to positive snow-albedo feedback processes (e.g., Alexander et al. 2010). Similar to s_macro_ T_0 − 500 m_, s_macro_ TS generally lies within the 2.5th -97.5th percentile range of the Pictl distribution during the first 3 decades (indicated by the lack of contours), with some localized exceptions (Fig. 3a-c). Note that even in the first decade over the SPNA and Arctic, s_macro_ TS falls mostly within the Pictl range, unlike s_macro_ T_0 − 500 m_, which showed significantly reduced values compared to the Pictl. The 4th decade exhibits significantly enhanced s_macro_ TS over a widespread area of the tropical Pacific and subtropical southwest Pacific compared to the Pictl distribution (contours in Fig. 3d), similar to the results for s_macro_ T_0 − 500 m_. Portions of the north and south Pacific (and south Atlantic) exhibit significantly enhanced s_macro_ TS in the 5th -6th decades compared to the Pictl distribution, while the SPNA and Pacific show significantly reduced s_macro_ TS in the 6th decade (contours in Fig. 3e-f).

The fractional changes in TS ensemble spread due to macro vs. micro initialization (s_frac_) show statistically significant, positive values in the first decade over the subpolar and northwest tropical Atlantic, subpolar North Pacific, tropical Indian Ocean and portions of the Southern Ocean, with values ranging from 30 to 80% (Fig. 3g). In the 2nd decade, significant fractional changes are mainly confined to the Arctic and SPNA (Fig. 3h). Beyond the 2nd decade, there is relatively little difference in ensemble spread due to macro vs. micro initialization with the notable exception of the western tropical Pacific in the 4th decade, which shows fractional differences around 30–60% (Fig. 3j); the equatorial Atlantic and SPNA also show significant negative fractional changes of 30–40% in the 5th and 6th decades, respectively (Fig. 3k).

**Fig. 4:**
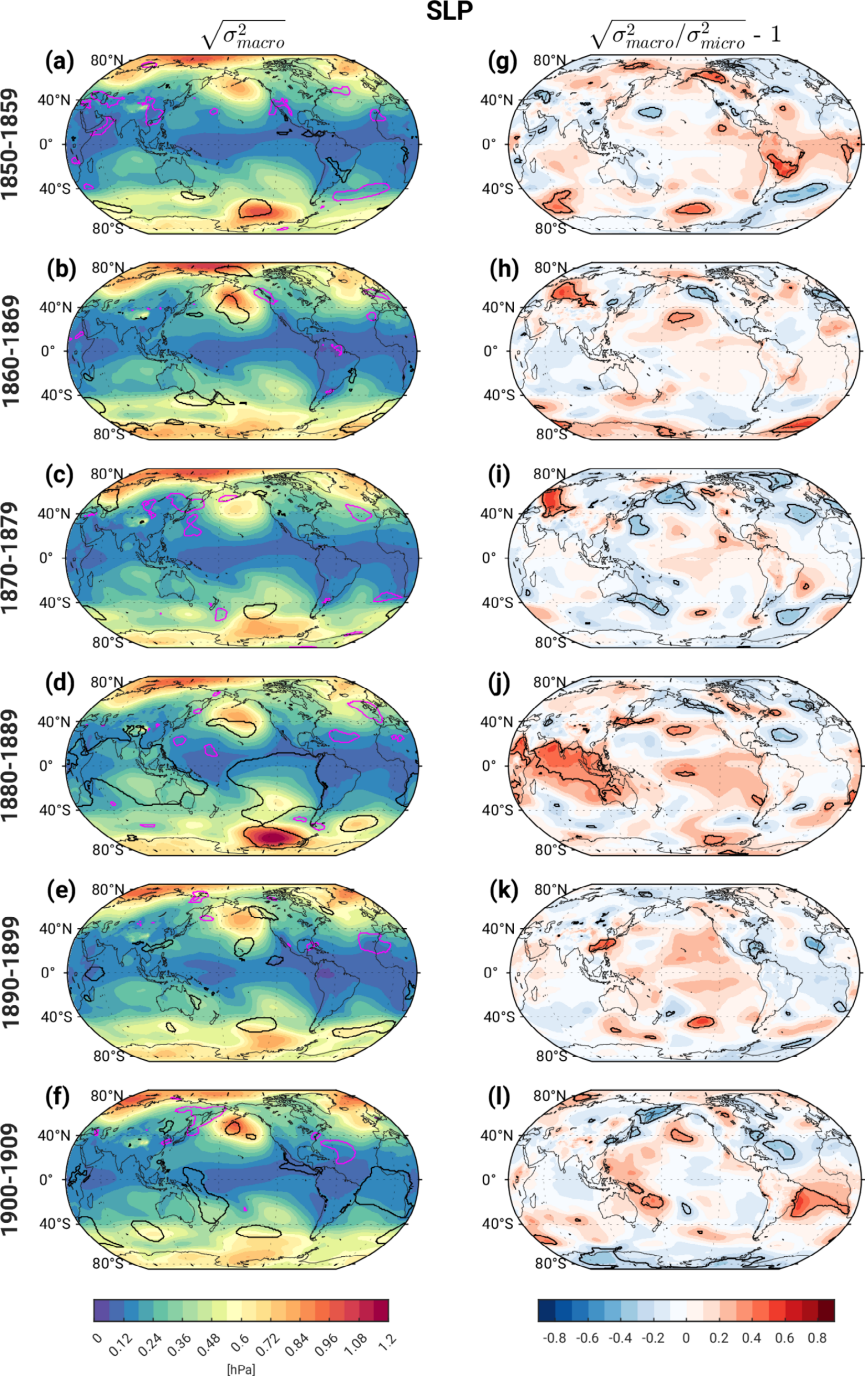
As in Fig. 2 but for sea level pressure (SLP)

The magnitudes of s_macro_ SLP increase with latitude as expected from geostrophy, with regional maxima over the North and South Pacific, and secondary maxima over the tropical South Pacific and Indian Oceans (Fig. 4a-f). These regional features are associated with the well-known Aleutian and Amundsen Sea Low Pressure Systems and the Southern Oscillation, respectively. While the amplitudes of these regional features vary from decade to decade, s_macro_ SLP generally lies within the 2.5th – 97.5th % spread diagnosed from the Pictl distribution, with the striking exception of the 4th decade when most of the tropical Indian and eastern Pacific Ocean (along with the Aleutian and Amundsen Sea Lows) shows significantly enhanced s_macro_ SLP compared to the Pictl distribution (Fig. 4d); significantly enhanced s_macro_ SLP is also seen over the tropical South Atlantic in the 6th decade (Fig. 4f). Unlike T_0 − 500 m_ and TS, SLP ensemble spread shows little sensitivity to the method of initialization (macro vs. micro) in the first decade, apart from a few isolated locations (Fig. 4g). The 4th decade shows the most widespread occurrence of statistically significant differences in s_macro_ SLP vs. s_micro_ SLP, with fractional values ~ 30–50% over the tropical Indian Ocean (Fig. 4j).

**Fig. 5:**
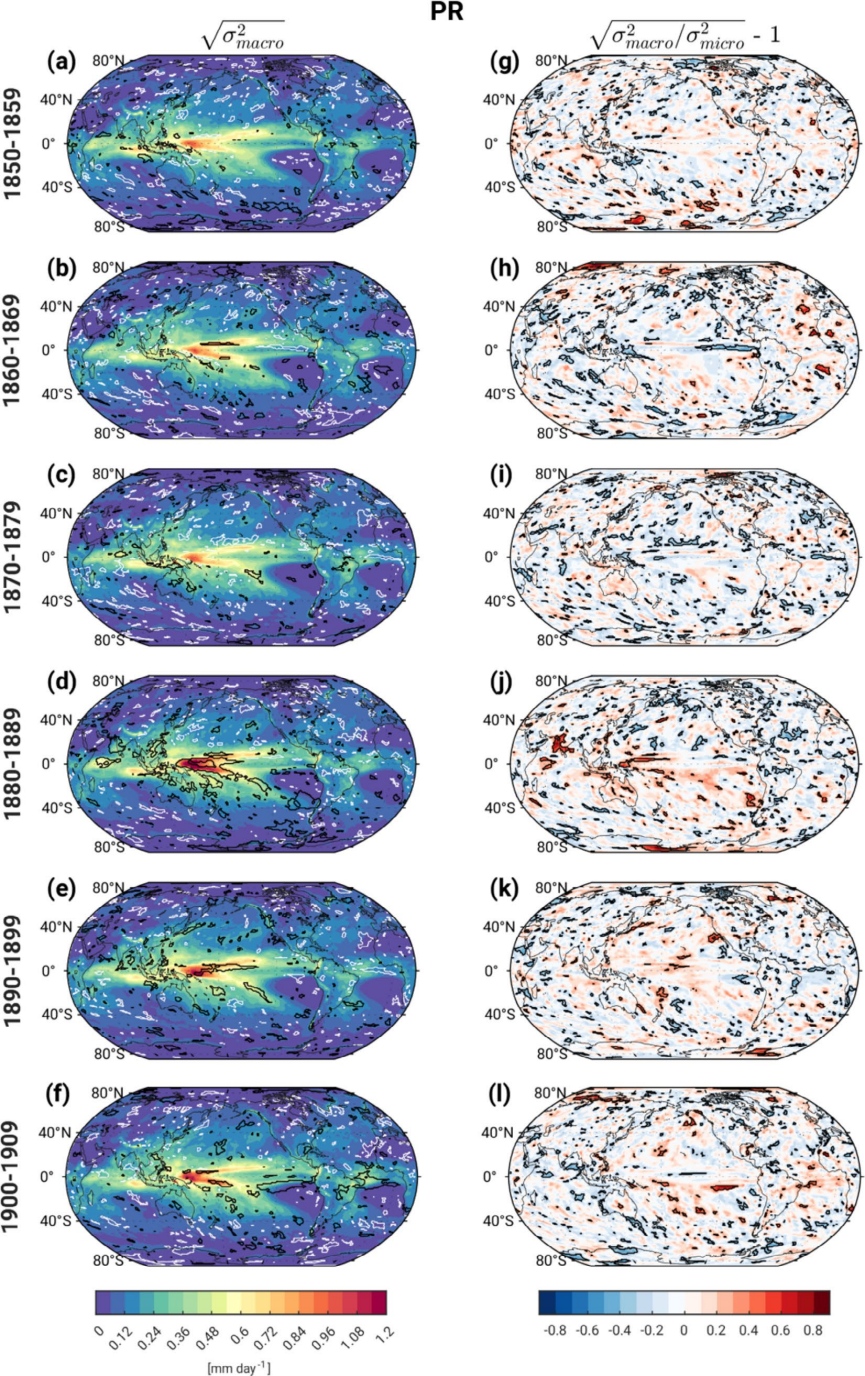
As in Fig. 2 in but for precipitation (PR). For readability, white contours are used in place of magenta in the left column

The magnitudes of s_macro_ PR are greatest in areas of tropical deep convection, where the mean climatological precipitation is greatest, with maximum values in the western equatorial Pacific (Fig. 5a-f). This pattern exhibits a notable amplification in the 4th decade that persists (with some damping) over the following two decades especially over the tropical western Pacific, similar to the temporal evolution of s_macro_ T_0 − 500 m_, TS and SLP. It is challenging to assess the degree to which s_macro_ PR is significantly influenced by external forcing due to the noisy appearance of the contours in Fig. 5a-f. However, coherent patches of s_macro_ PR > 97.5th % Pictl over the western tropical Pacific and parts of the tropical Indian Ocean in the 4th decade (black contours in Fig. 5d) and to a lesser extent the 5th decade (black contours in Fig. 5e) are plausibly physically linked to analogous features in s_macro_ TS and s_macro_ SLP (recall Figs. 3d-e and 4d-e, respectively) and are thus more likely to be externally-forced. In contrast, the small areas contoured in white in Fig. 5a-f probably arise solely by chance. The maps of fractional change s_frac_ in PR ensemble spread also exhibit considerable small-scale noise, with limited areas of coherent statistical significance (Fig. 5g-l). However, significant values of 30–70% over the Arabian Sea and far western equatorial Pacific in the 4th decade correspond to regions identified as having statistically significant fractional changes in TS and SLP, lending support to their physical validity.

**Fig. 6:**
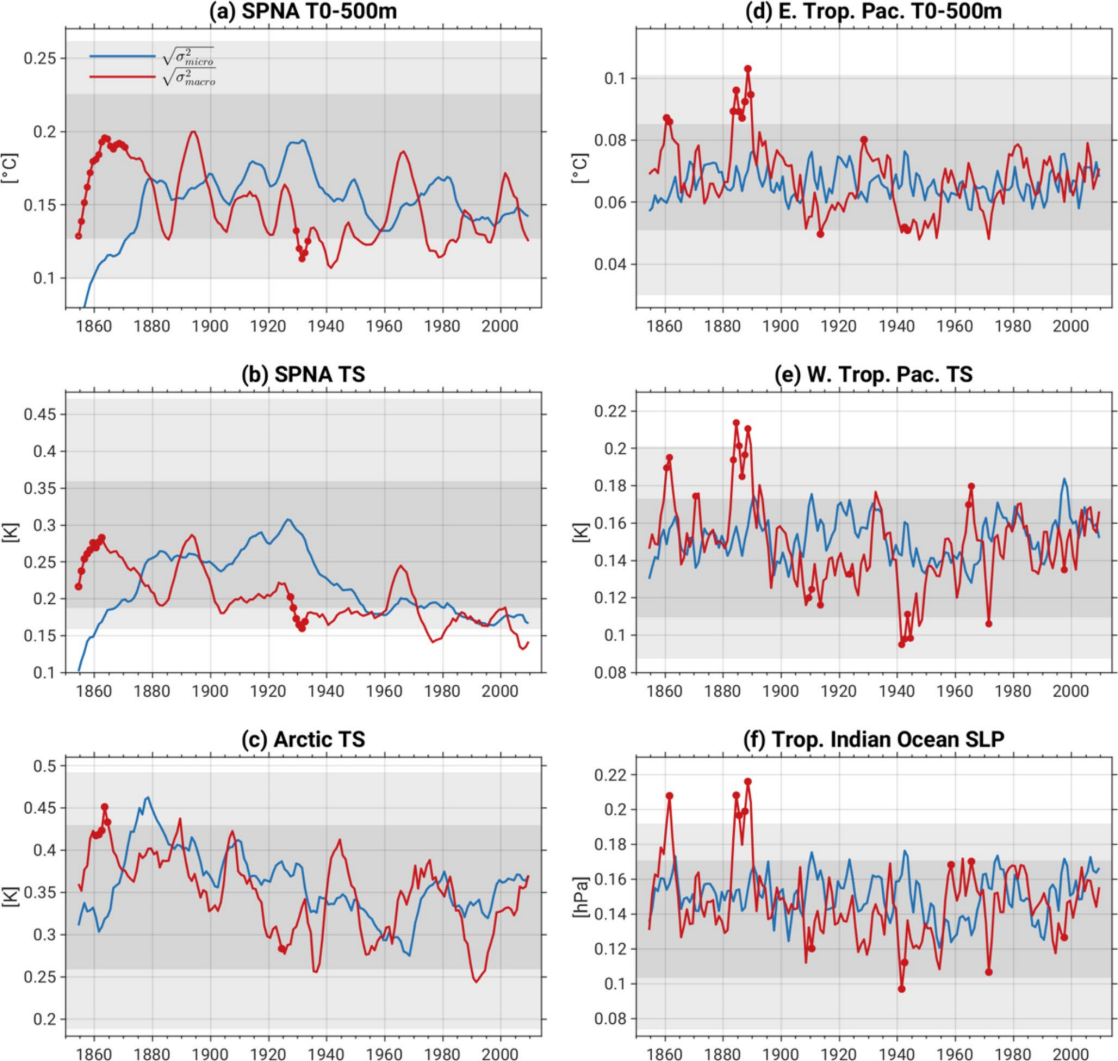
Ensemble spread due to macro (red curves) and micro (blue curves) initialization for 10-year running means of: (**a**) Subpolar North Atlantic (SPNA: 45°-65°N, 50°W-0°) T_0 − 500 m_, (**b**) SPNA TS, (**c**) Arctic (67°-90°N) TS, (**d**) eastern tropical Pacific (15°S-5°N, 145°-100°W) T_0 − 500 m_, (**e**) western tropical Pacific (140°E-140°W, 12°S-7°N) TS and (**f**) Tropical Indian Ocean SLP (40°E-140°E, 20°S-20°N). Red circles on the red curves indicate that the spread due to macro-initialization is significantly different from that due to micro-initialization. Darker (lighter) gray shading denotes the 2.5th – 97.5th percentile (full) range diagnosed from the Pictl simulation

A complementary view of ensemble spread behavior produced by macro vs. micro initialization is provided in Fig. 6, which shows timeseries of ensemble spread from 1850 to 2015 for a selection of regional 10-year running mean indices. The choice of these indices is motivated by the ensemble spread maps shown above. Statistically significant differences between the macro (red curves) and micro (blue curves) ensemble spread timeseries are indicated with red circles (on the red curves), and the dark (light) gray shading denotes the 2.5th – 97.5th percentile (full) range of ensemble spread diagnosed from the Pictl simulation. The SPNA (45°-65°N, 50°W-0°) T_0 − 500 m_ and TS indices exhibit significantly larger spread with macro- compared to micro- initialization over the first 20 and 15 years of the simulation, respectively, and coincident with micro-initialization ensemble spread falling below the 2.5th % range diagnosed from the Pictl (dark gray shading in Fig. 6a, b). Beyond this time frame, SPNA ensemble spread is statistically comparable between the two initialization methods (with the exception of a few years centered around 1930 when, surprisingly, s_micro_ exceeds s_macro_), and lies nearly always within the Pictl distribution (albeit in the lower half of the Pictl distribution). Ensemble spread in Arctic (north of 67°N) TS is significantly larger with macro compared to micro initialization from 1859 to 1866, but otherwise exhibits no notable contrasts and lies well within the Pictl spread throughout the historical record (Fig. 6c). An overall decline in s_macro_ from the 1860–1940 s is evident in the SPNA and Arctic indices. All three tropical indices [eastern Tropical Pacific (15°S-5°N, 145°-100°W) T_0 − 500 m_, western Tropical Pacific (140°E-140°W, 12°S-7°N) TS and Tropical Indian Ocean (40°E-140°E, 20°S-20°N) SLP] exhibit significantly enhanced ensemble spread with macro compared to micro initialization during the 1880s (and more briefly around 1860; Fig. 6e-f). During these intervals, spread due to macro initialization exceeds the 97.5th percentile of the Pictl distribution, suggestive of a role for external forcing. In the early 1940s, s_macro_ fall significantly below s_micro_ and lies near the 2.5th % of the Pictl. Apart from these signals, no significant deviations in ensemble spread occur between macro vs. micro initialization, nor between either initialization method and the Pictl simulation, except for a few isolated years.

In summary, the evolution of ensemble spread in the set of CESM2 historical simulations examined here exhibits limited sensitivity to the method of initialization (e.g., micro vs. macro perturbations) beyond the first two decades, with the notable exception of the 1880s. This sensitivity is primarily confined to T_0 − 500 m_ and TS over the SPNA and Arctic, with spread due to macro-initialization significantly exceeding that due to micro-initialization by 30–80% depending on location. These fractional differences result mainly from significant suppression of micro-perturbation ensemble spread compared to the Pictl simulation (recall Fig. 6). An unexpected resurgence of statistically significant macro vs. micro ensemble spread differences appears in the 4th decade (1880–1889) over the tropical Indo-Pacific in all four variables examined, with fractional values ranging from 30 to 60%. We speculate that this coordinated signal may be indicative of a state-dependent coupled tropical ocean-atmosphere response to the large sequential volcanic eruptions of Krakatoa (located in Indonesia) in 1883 and Mt. Tarawera (located in northern New Zealand) in 1886. In particular, the temporal and spatial proximity of the dual eruptions to the occurrence of significant macro vs. micro ensemble spread differences hints at a causal link. While this conjecture remains to be tested, we note that a state-dependent response to idealized tropical volcanic eruptions has been reported in previous modeling studies (e.g., Zanchettin et al. 2022 and 2023). That such a state-dependent response is preferentially manifest in the set of macro-initialized simulations may reflect their wider range of oceanic states at the time of the eruptions compared to the micro-initialized ensembles. In Section 4b, we provide circumstantial evidence in support of this conjecture; however, further investigation is needed to directly test this hypothesis and understand the mechanisms underlying the state-dependent response.

Apart from these notable regional signals, the evolution of ensemble spread over the historical period 1850–2000 lies generally within the range diagnosed from the Pictl simulation regardless of initialization method, including globally averaged T_0 − 500 m_ and TS (Fig. S3). As such, our results contrast with the findings of Hawkins et al. (2016) who reported a decline in ensemble spread in global (and European) TS from approximately years 20–100 of their 1% per year CO_2_ simulations in both their macro and micro initialization ensembles; however, they did not have an unforced control simulation to compare against, so the significance of this decline could not be assessed. The increase in global T_0 − 500 m_ ensemble spread over the second half of the 20th century and its exceedance relative to the likely (2.5th – 97.5th percentile) Pictl range (Fig. S3) are consistent with the findings of Stevenson et al. (2023), although they were not able to separate the impacts of initialization method from model structural uncertainty as discussed earlier.

### Ocean initial-condition memory effects on ensemble spread

**Fig. 7:**
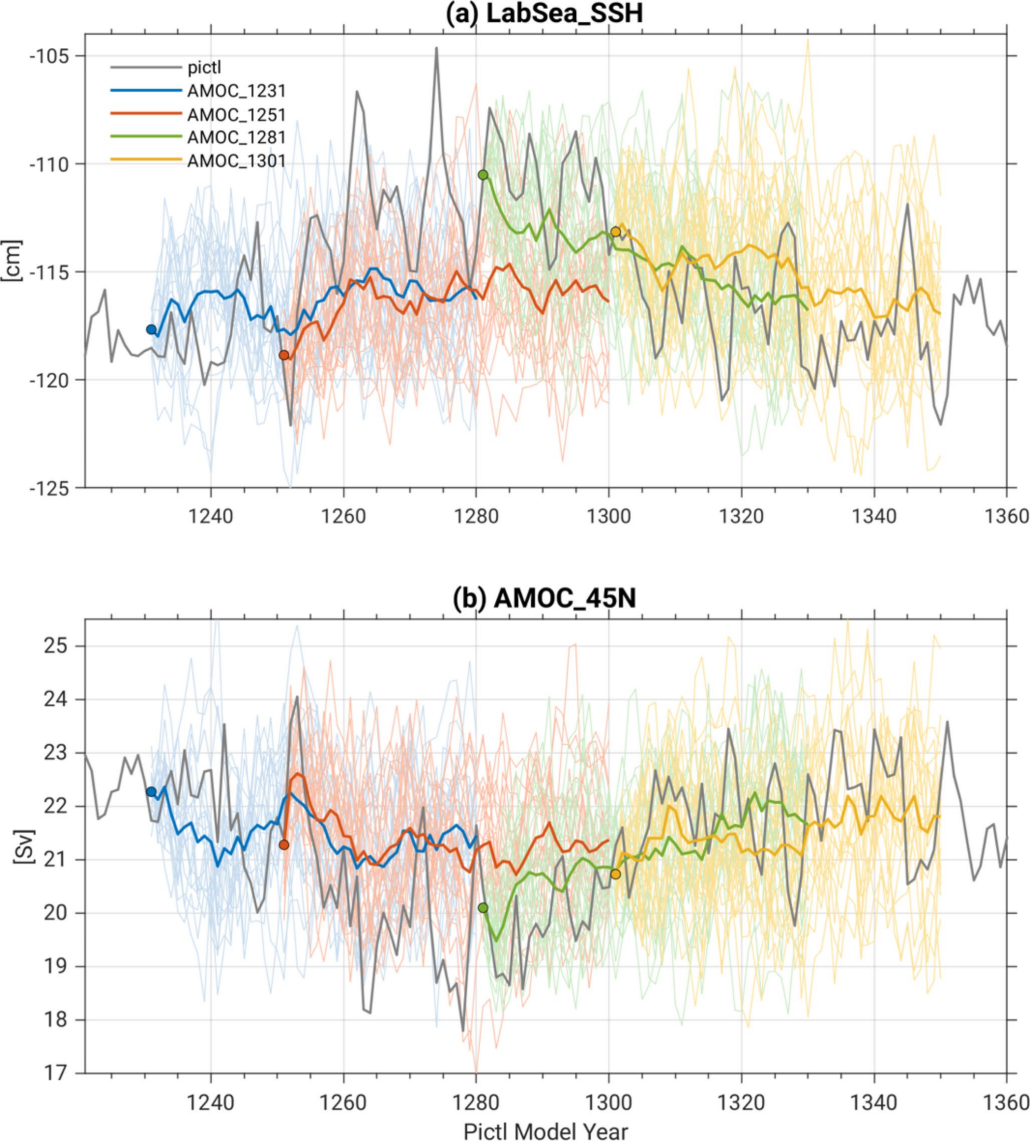
Annual timeseries of (**a**) LabSeaSSH and (**b**) AMOC45N from the CESM2 pre-industrial control simulation (gray) and the AMOC1231, AMOC1251, AMOC1281 and AMOC1301 ensembles (thin colored curves for each ensemble member, thick curves for ensemble means; colored circles mark the ensemble means for the first year)

In this section, we investigate ocean initial-condition memory effects on the evolution of ensemble spread based on the four 20-member AMOC ensembles. Recall that the initial conditions were chosen to sample different phases of a prominent centennial-scale AMOC oscillation in the Pictl simulation during years 1231–1301 (Fig. 1). The LabSeaSSH and AMOC45N timeseries in the first 50 years of each historical AMOC ensemble are compared with the Pictl in Fig. 7. As expected, the Pictl (gray curve) generally lies within the spread of each AMOC ensemble (thin colored curves). However, the ensemble-mean timeseries in AMOC1231 and AMOC1251 (thick blue and red curves, respectively) exhibit considerably weaker trends compared to the Pictl, while those in AMOC1281 and AMOC1301 (thick green and yellow curves, respectively) show trends that are comparable to those in the Pictl. While it is tempting to interpret this result as reflecting a state-dependence of long-term (e.g., 4–5 decades ahead) AMOC predictability, external forcing produces a small (0.5 Sv) increase in AMOC strength over the first 50 years of the historical simulations (Fig. 1f in Rodgers et al. 2021), which is enough to reconcile the magnitudes of the predictable component of internally-generated trends in the four AMOC ensembles.

**Fig. 8:**
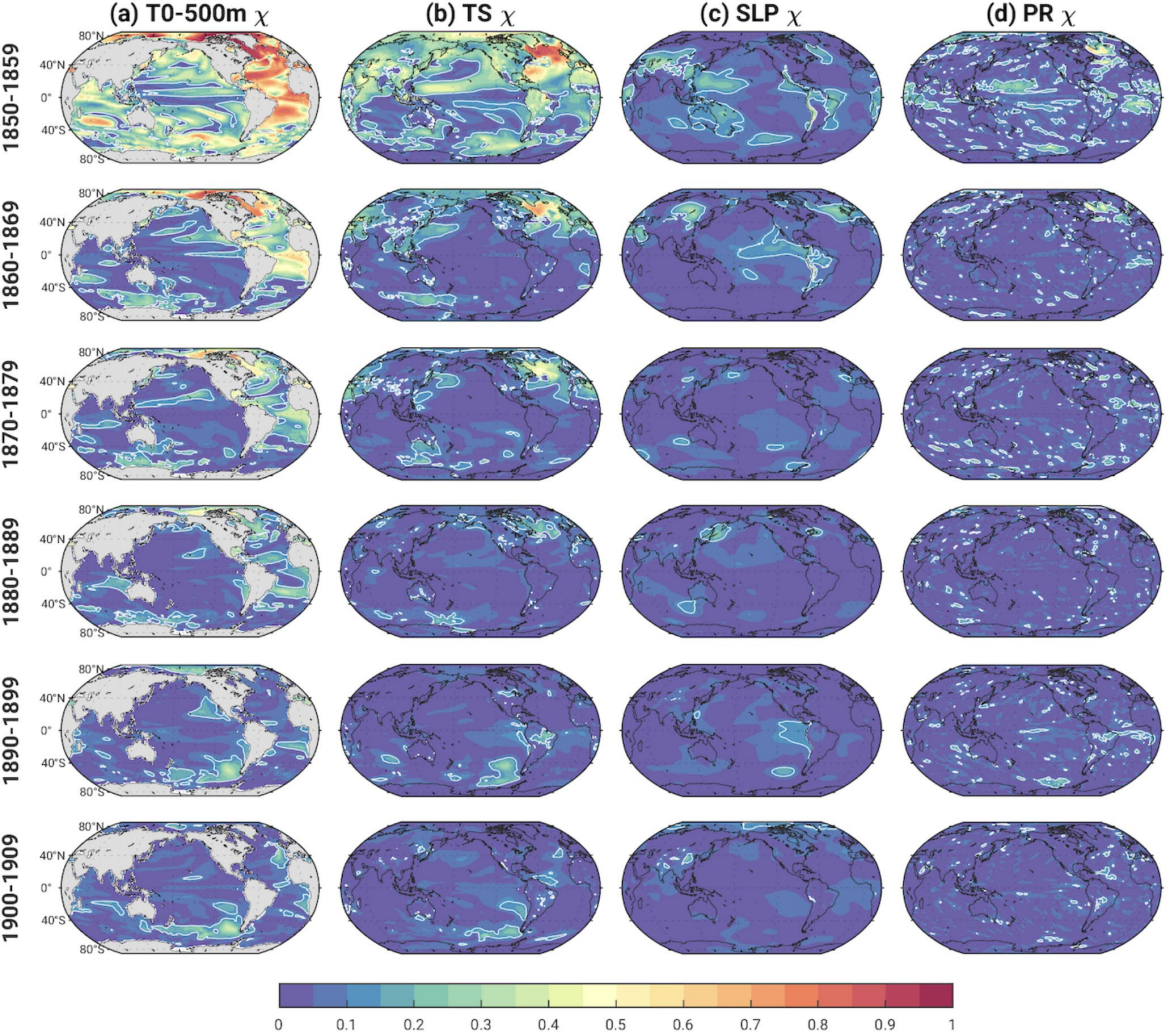
Fraction of ensemble spread attributable to ocean initial-condition memory in the 4 AMOC ensembles [χ_ocean_ = (s^2^_ocean_/s^2^_total_)] based on decadal-means over the first 6 decades of the historical simulations. (Left to right): T_0 − 500 m_, TS, SLP and PR. White contours outline regions that are statistically significant at the 95% confidence level based on bootstrapping the Pictl simulation (see text for details)

Figure 8 shows the evolving fraction of ensemble spread (variance of decadal means) attributable to ocean initial-condition memory χ_ocean_ (defined as s^2^_ocean_/s^2^_total_ : see Eq. 1) in the four 20-member AMOC ensembles over the first 6 decades of the historical simulations for T_0 − 500 m_, TS, SLP and PR. Statistical significance of χ_ocean_ is determined from the Pictl simulations as follows. We randomly sample 80 decadal averages from years 800–1599 of the de-drifted Pictl, divide them into four groups of 20 and average the 20 values in each group, compute the ratio of the variance across the four sets of 20-member averages divided by the variance across the 80 members, and repeat this entire procedure 3000 times to identify the 2.5th − 97.5th percentile range of this ratio that could occur by chance (e.g., due to sampling fluctuations). Values of χ_ocean_ that lie outside this range are considered statistically significant (i.e., attributable to ocean initial-condition memory) and marked by contours in Fig. 8.

In the first decade, significant values of T_0 − 500 m_ χ_ocean_ are widespread, with the largest values (exceeding 0.8) over the Atlantic basin and the Arctic (Fig. 8a). Significant values persist through the 4th decade over the Atlantic sector, especially the western SPNA and Canadian Arctic, albeit with a gradual decline in magnitude. Significant long-lasting T_0 − 500 m_ χ_ocean_ is also found over the Southern Ocean, persisting into the 1880s between Antarctica and Australia, and subsequently appearing in the 1890–1900 s in the Pacific sector with maximum values around 0.5.

TS shows long-lasting ocean initial-condition memory over the SPNA, the Arctic and a broad region encompassing Europe, the Mediterranean and North Africa, with statistically significant χ_ocean_ extending into the 3rd and 4th decades in these regions (Fig. 8b). Not surprisingly, the SPNA exhibits the largest TS χ_ocean_, with values exceeding 0.9 in the first decade and dropping to about 0.3 in the 4th decade. This spatio-temporal pattern of ocean initial-condition memory is typical of that associated with AMOC fluctuations (e.g., Kim et al. 2018; Wills et al. 2019; Zhang et al. 2019) and is investigated further below. Like T_0 − 500 m_, TS exhibits a coherent region of significant χ_ocean_ over the Southern Ocean between Australia and Antarctica that appears to shift eastward over the first four decades. Subsequent development of statistically significant χ_ocean_ over the eastern Pacific sector of the Southern Ocean is found in the 5th and 6th decades, extending into the subtropical eastern Pacific in the 6th decade.

Compared to TS and T_0 − 500 m_, SLP and PR exhibit weaker, less persistent and less widespread significant ocean initial-condition memory, with maximum values of χ_ocean_ generally < 0.3 in the first two decades (Fig. 8c-d). In particular, significant χ_ocean_ is found over the SPNA, western subtropical Pacific and parts of the tropical Atlantic and Africa in the first decade and persists over the SPNA (and equatorial Atlantic for PR) into the second decade; SLP also shows significant χ_ocean_ over the equatorial Pacific and northern China in the second decade. Significant initial-condition memory is virtually absent in the 3rd and 4th decades, but, curiously, reappears in the 5th decade in SLP over the eastern tropical Pacific and in PR over the equatorial Atlantic (and in both variables over the Amundsen Sea). This resurgence of significant χ_ocean_ is likely associated with similar features in TS χ_ocean_.

**Fig. 9:**
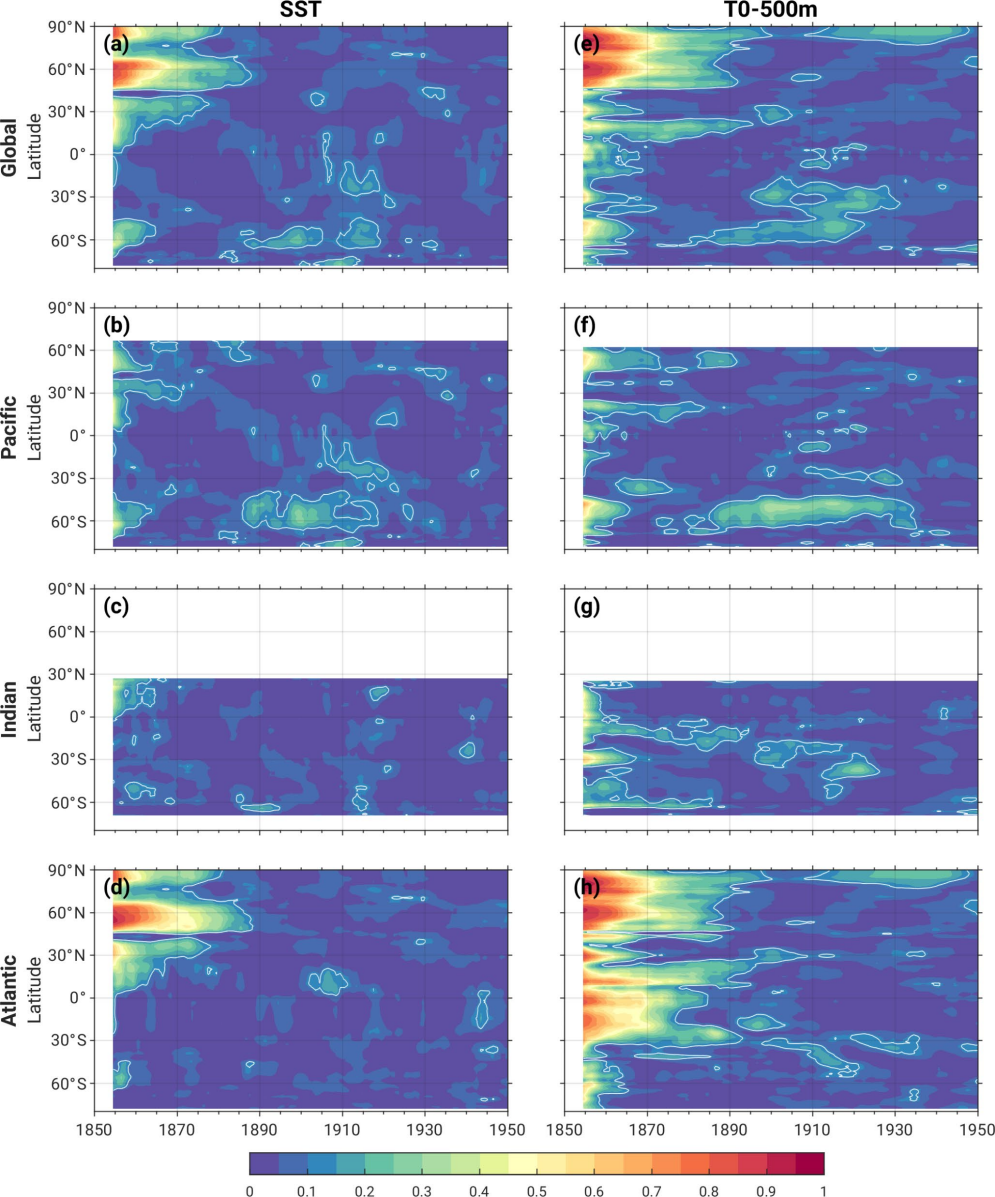
Zonal-mean Hovmöller diagrams of the fraction of ensemble spread of 10-yr running mean (left) SST and (right) T_0 − 500 m_ attributable to ocean initial-condition memory in the 4 AMOC ensembles [χ_ocean_ = (s^2^_ocean_/s^2^_total_)] for the following domains: **a**,**e**) global; **b**,**f**) Pacific; **c**,**g**) Indian; and **d**, **h**) Atlantic. White contours outline regions that are statistically significant at the 95% confidence level based on bootstrapping the Pictl simulation (see text for details)

To further highlight the temporal evolution of ocean initial-condition memory, we construct Hovmöller diagrams of χ_ocean_ based on zonally averaged timeseries of T_0 − 500 m_ and sea surface temperature (SST: ocean model output variable “tos”) for the globe and for the Pacific, Indian, and Atlantic sectors (Fig. 9). In the global average, SST χ_ocean_ maximizes primarily in the extratropical Northern Hemisphere over the first 3–4 decades, with significant values peaking in the latitude bands 10°-40°N, 45°-65°N, and 80°-90°N (Fig. 9a). These features arise primarily from the Atlantic sector (Fig. 9d), as expected based on the choice of initial ocean states and the decadal predictability of Atlantic SSTs (Yeager et al. 2018; Smith et al. 2019; Yeager 2020). The high latitude Southern Hemisphere also exhibits significant global-mean SST χ_ocean_ in the first decade and again from the mid-1880s through the end of the 1920s, with peak values in the latitude band 45°-70°S (Fig. 9a). A secondary maximum occurs at lower latitudes (15°-30°S) from the mid-1900s through the early 1920s (Fig. 9a). These Southern Hemisphere features originate primarily in the Pacific sector (Fig. 9b).

Analogous behavior is seen for T_0 − 500 m_ χ_ocean_, with even longer duration and higher amplitude of significant χ_ocean_ compared to SST (Fig. 9e-h). This is particularly evident over the Atlantic sector, where significant T_0 − 500 m_ χ_ocean_ lasts for 4–5 decades, not only in the northern extratropics but also in the tropics (Fig. 9h). The Pacific sector of the Southern Ocean is another region of pronounced T_0 − 500 m_ χ_ocean_, with significant values in the latitude band 45°-65°S in the first decade and again from the 1880–1930 s (Fig. 9f). The Indian sector displays a southward progression of significant T_0 − 500 m_ χ_ocean_ from the Equator to about 40°S from the 1880–1920 s, although the values are relatively weak (< 0.15) and not continuous (Fig. 9g).

In summary, there appear to be two distinct stages of statistically significant ocean initial-condition memory in SST and T_0 − 500 m_ across the four AMOC ensembles: an initial stage focused in the North Atlantic that lasts for approximately 3–4 and 4–5 decades, respectively; and a second stage that appears ~ 35 years later (e.g., ~ 1885) in the Pacific sector of the Southern Ocean and subsequently in the subtropical southeast Pacific in ~ 1905 and lasts through the 1920s and 1930s, respectively.

**Fig. 10:**
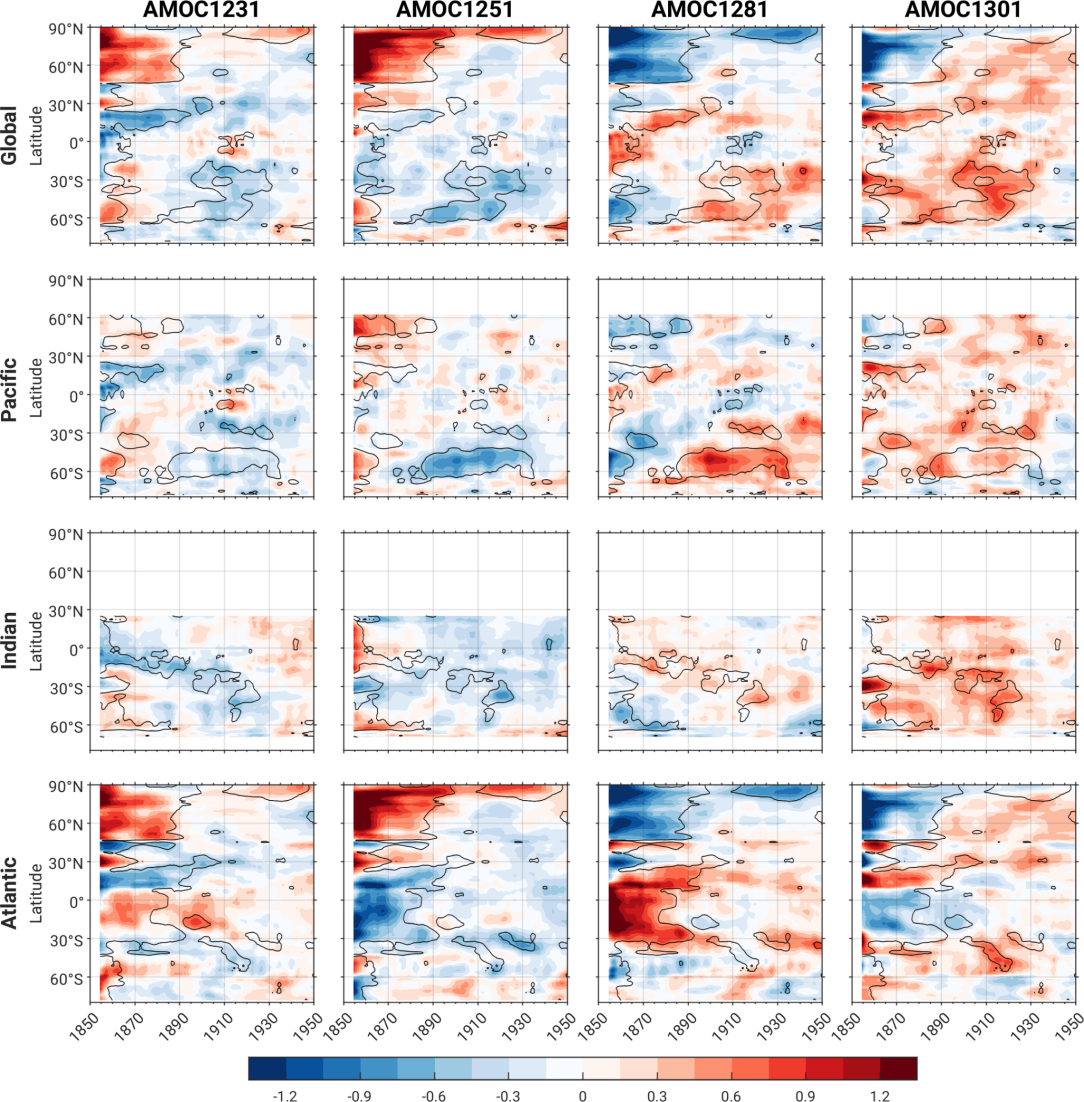
Hovmöller diagrams of zonally averaged ensemble-mean T_0 − 500 m_ normalized anomalies for each AMOC ensemble based on 10-year running means for the following domains: (top row) global; (2nd row) Pacific; (3rd row) Indian; and (bottom row) Atlantic. Anomalies are defined relative to the average of the 4 AMOC ensemble-means and normalized by the de-drifted Pictl standard deviation. Contours of statistically significant χ_ocean_ from Fig. 9 are superimposed for context

Next, we examine the characteristics of initial-condition memory in each AMOC ensemble individually, since they may not necessarily follow the same evolution given their different starting points (recall Fig. 7). Hovmöller diagrams of 10-year running mean timeseries of zonally averaged ensemble-mean T_0 − 500 m_ anomalies for each individual AMOC ensemble are shown in Fig. 10, with statistically significant χ_ocean_ taken from Fig. 9 superimposed for context (analogous diagrams for SST are shown in Fig. S4). To compute these anomalies, we subtracted the average of the 4 AMOC ensemble-means from each individual AMOC ensemble-mean, and then normalized the anomalies by the Pictl standard deviation (based on detrended data during years 800–1599) to give a sense of their relative amplitudes. For zonal averages across the Pacific sector, it is clear that AMOC1251 and AMOC1281 make the largest contribution to the statistically significant values of χ_ocean_ in the latitude band 40-65°S (Fig. 10). In this region, AMOC1251 (AMOC1281) exhibits positive (negative) anomalies of up to 0.7 standard deviations in the first decade, transitioning to negative (positive) anomalies of similar magnitude from about 1885–1935. These AMOC ensembles also exhibit contrasting Atlantic signatures, with large-amplitude anomalies (0.5–1.5 standard deviations) of opposite polarity extending over the first 40 years of the simulations, i.e., negative (positive) anomalies in the tropics and positive (negative) anomalies in the northern extratropics for AMOC1251 (AMOC1281) corresponding to the statistically significant values of χ_ocean_. While the North Atlantic anomalies in AMOC1231 and AMOC1301 resemble their respective AMOC1251 and AMOC1281 counterparts, the tropical South Atlantic anomalies differ in sign. Whether the distinctive tropical South Atlantic initial-condition memory during 1850–1890 in AMOC1251 and AMOC1281 compared to AMOC1231 and AMOC1301 is causally linked to the initial-condition memory that appears in the late 1880s in the South Pacific remains to be investigated.

It is also possible that the initial-condition memory signatures in the midlatitude South Pacific are independent of those in the Atlantic. That is, although the Pictl years selected for generating the four AMOC ensembles are based on AMOC45N and LabSeaSSH, they will also sample different Southern Ocean states which may themselves give rise to long-lived anomalies. While it is beyond the scope of the current study to address the physical causes of initial-condition memory in the Pacific sector of the Southern Ocean, we note that AMOC1251 and AMOC1281 both exhibit appreciable ensemble-mean T_0 − 500 m_ anomalies between Australia and East Antarctica in the first four decades of the simulations, with anomalies of opposite sign developing directly to the east in the 3rd decade and expanding across the entire Pacific sector of the Southern Ocean by the 5th to 6th decades (Fig. S5).

## Discussion

### Mechanisms of initial-condition memory related to AMOC

Here, we investigate the mechanisms underpinning the first stage of ocean initial-condition memory in the North Atlantic in the four AMOC ensembles. As discussed above, the long-lived (up to 5 decades) initial condition memory in T_0 − 500 m_ and SST over the SPNA and Arctic is a notable feature that can be attributed, by design, to the initial condition memory associated with different AMOC states. A mechanism that maintains long memory in the SPNA was first proposed by Yeager (2020) using CESM1 and has since been confirmed with multiple models including CESM2 (e.g., Kim et al. 2024) and CESM1 at high-resolution (Yeager et al. 2021) as well as in observational data (Chafik et al. 2023). In essence, this mechanism attributes the source of the upper SPNA thermal memory to the deep, denser limb of AMOC and its interaction with the upper, lighter limb of AMOC. In the SPNA, strong surface buoyancy forcing (e.g., associated with the North Atlantic Oscillation) generates anomalous deep, dense waters. Although a part of these waters propagates relatively quickly along the deep western boundary current, a bulk of the dense waters propagates more slowly to the south through the interior of the western SPNA (Bower et al. 2011) and is discharged into the subtropics west of the Mid-Atlantic Ridge (MAR). This anomalous dense water can generate a zonal sea surface height (SSH) gradient around the MAR through the steric effect. The zonal SSH gradient drives anomalous geostrophic meridional flow that projects onto the North Atlantic Current (NAC), the major constituent of the upper AMOC limb that transports heat northward from the subtropics to the SPNA. Because the SSH anomaly spins up the entire subpolar gyre, the anomalous NAC is largely canceled by an anomalous Labrador Current (the western boundary current flowing southward along the Labrador coast) when the flows are integrated zonally. Therefore, the signature of an anomalous NAC is obscured in AMOC when depicted in conventional depth coordinates. On the other hand, because the anomalous NAC is lighter than the Labrador Current, its signature is more evident in AMOC depicted in density coordinates [AMOC(σ); Yeager 2020].

**Fig. 11:**
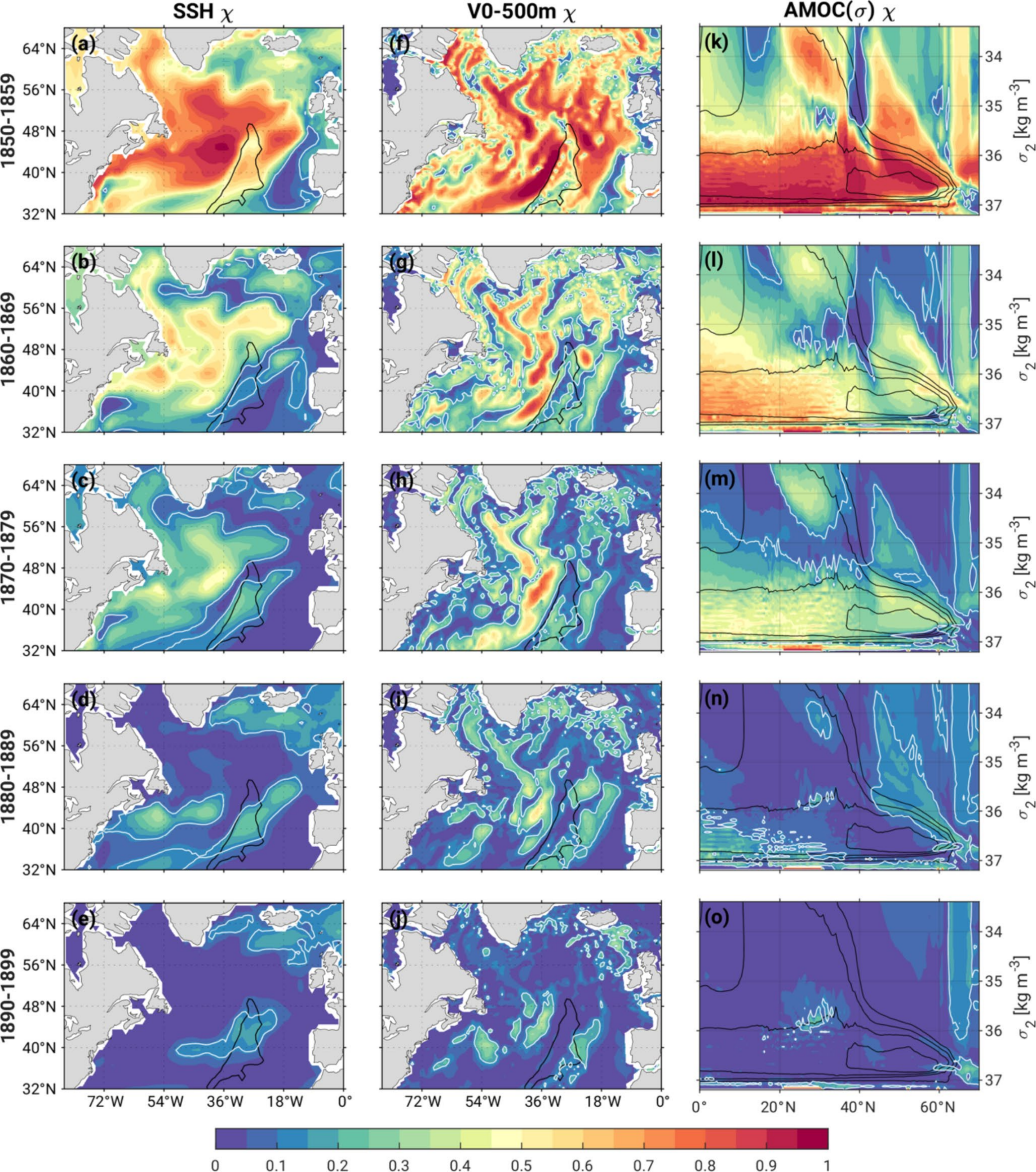
As in Fig. 8 but for χ_ocean_ of (**a**-**d**) sea surface height (SSH), (**f**-**j**) northward transport averaged over the upper 500 m of the ocean (V_0 − 500 m_), and (**k**-**o**) AMOC in density coordinates [AMOC(σ); the y-axis coordinate σ_2_ refers to potential density referenced to 2000 m relative to 1000 m). The decade 1900–1909 has been omitted for visual clarity. The black contour in the left and middle column outlines the Mid-Atlantic Ridge (MAR). The black contours in the right column represent the climatological AMOC(σ) in the Pictl (contour interval of 5 Sv starting from 5 Sv)

Consistent with this hypothesis, AMOC(σ) $\:{\chi\:}_{ocean}$ shows significant memory, lasting up to 4 decades, in its upper, lighter limb in the SPNA where it flows northward along σ_2_ layers lighter than 36.6 kg m^− 3^ (σ_2_ refers to potential density referenced to 2000 m) and in its denser limb that flows southward along σ_2_ layers denser than 37.0 kg m^− 3^ (Fig. 11k-n). While significant AMOC(σ) $\:{\chi\:}_{ocean}$ is found over much of the latitude-density space in the first two decades (Fig. 11k, l) significance is retained into the 4th decade only in these light and heavy density ranges north of 40°N (Fig. 11m, n). SSH $\:{\chi\:}_{ocean}$ shows a similar, long-lived memory in the western basin with the largest memory west of the MAR (located around 30°W) at the gyre boundary between the subtropics and SPNA around 45°N (Fig. 11a-e). This implies that the zonal SSH gradient and, consequently, upper ocean (0–500 m) meridional flow (V_0 − 500 m_) also exhibits extended initial-condition memory (Fig. 11f-j). In particular, significant V_0 − 500 m_$\:{\chi\:}_{ocean}$ shows maximum values near the MAR that last up to 5 decades (Fig. 11f-j). Similar results are found for northward heat transport (not shown). Thus, volume transport is the dominant factor giving rise to the long-lived initial-condition memory in SPNA upper-ocean temperatures in the four AMOC ensembles.

We note that the nearly 40-year AMOC memory found here is considerably longer than the 1–2 decades reported in previous studies (e.g., Griffies and Bryan 1997; Msadek et al. 2010; Branstator and Teng 2014). Several factors may contribute to this difference, including the amplitude and phase of the initial AMOC anomaly, model dependence, ensemble size and choice of metrics used to estimate predictability. As noted by Yeager (2020), the coordinate system used to define AMOC may affect its predictability characteristics. In particular, for the cases examined here, the initial-condition memory of the northward flowing upper AMOC limb at the intergyre boundary (~ 45°N) is reduced from ~ 4 decades when viewed in density coordinates (densities < 36.7 kg m^3^ ) to ~ 1 decade when viewed in conventional depth coordinates (depths < 1000 m; Fig. S6). We would like to emphasize that while most previous studies consider predictability of AMOC defined in depth coordinates, AMOC defined in density coordinates best connects to predictability of upper ocean temperatures in the SPNA.

### The potential role of volcanic eruptions on ensemble spread

**Fig. 12:**
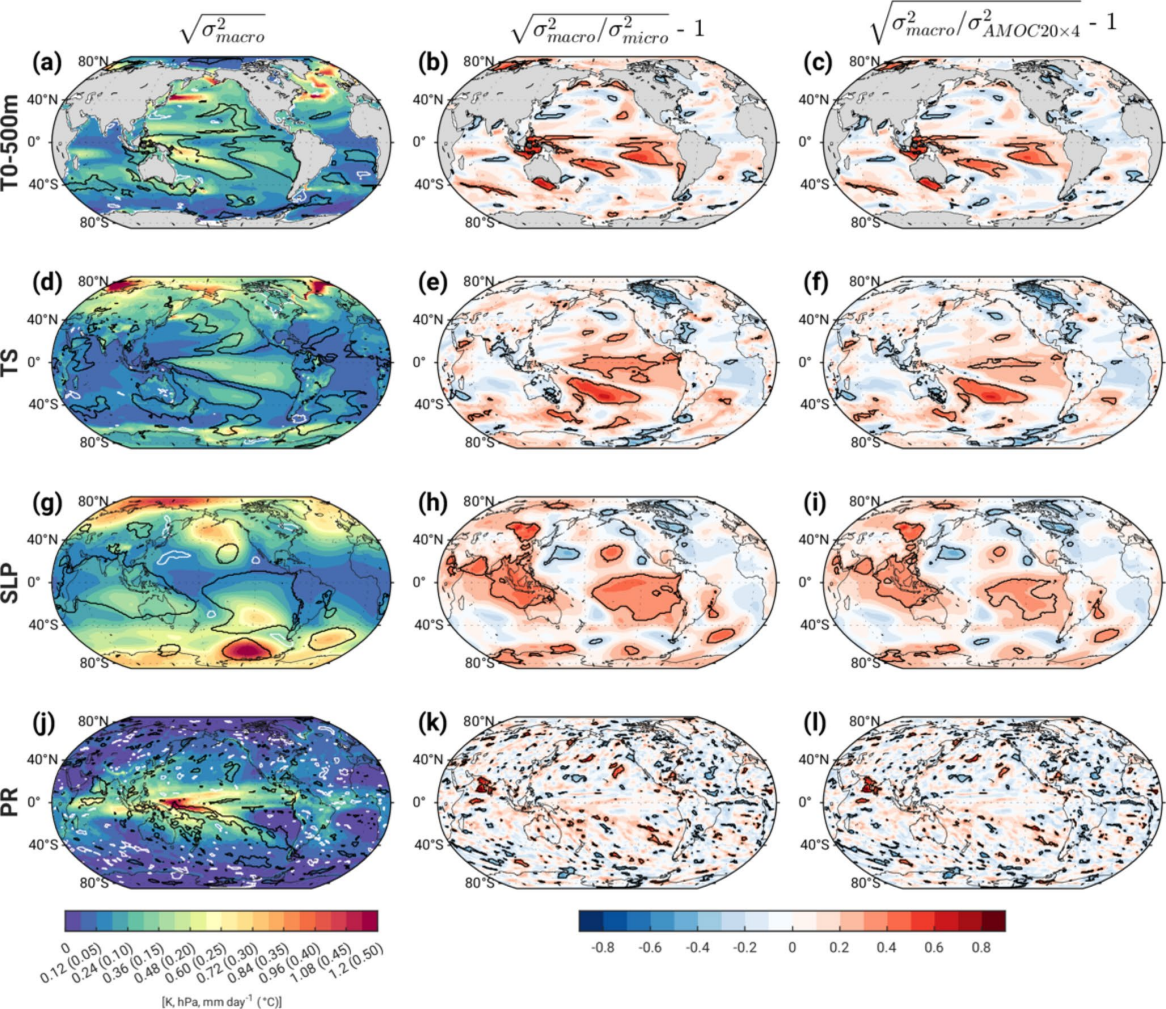
Ensemble spread for the decade 1884–1893 based on (left) macro-initialization (s_macro_), (middle) fractional difference between s_macro_ and s_micro_, and (right) fractional difference between s_macro_ and s_amoc4 × 20_. See text for definitions. The black (white) contours in the left column enclose regions where s_macro_ lies above (below) the 97.5th (2.5th ) percentile of the distribution in spread diagnosed from the Pictl. The black contours in the middle and right columns enclose regions where the fractional differences are statistically significant at the 5% confidence level based on bootstrapping the Pictl (see text for details)

In Section 3a, we speculated that the sequential volcanic eruptions of Krakatoa in May-Oct 1883 and Mt. Tarawera in June 1886, which together injected record amounts of sulfur dioxide into the stratosphere (see Fig. 5 in Danabasoglu et al. 2020), may have led to the significant enhancement of ensemble spread in the tropical Indo-Pacific sector during the 1880s in the macro-initialized simulations (s_macro_) compared to that diagnosed from the (unforced) Pictl and compared to that in the micro-initialized simulations (s_micro_). This conjecture relies on two untested assumptions: (1) CESM2 simulates a state-dependent response to these volcanic eruptions; and (2) the macro-initialized ensemble samples a broader range of relevant (tropical) oceanic states at the time of the eruptions compared to the micro-initialized ensemble. Without additional experiments, we cannot determine the nature of the state-dependent response to the eruptions, since a large number of ensemble members branched from each macro simulation at the start of the eruption sequence would be required to isolate it from internal variability. However, as a preliminary step toward addressing the second assumption, we have compared s_macro_ with s_micro_ and with the total spread across the 80 members of AMOC4 × 20 (denoted s_AMOC4 × 20_) based on decadal averages over 1884–1893 (the decade that is expected to be most relevant for a state-dependent response to the eruptions and their aftermath). The results confirm that s_macro_ T_0 − 500 m_ and s_macro_ TS significantly exceed the 97.5th % of the Pictl distribution and significantly exceed s_micro_ and s_AMOC4 × 20_ over substantial portions of the tropical Pacific (black contours in Fig. 12a-f). Likewise, the results confirm that s_macro_ SLP significantly exceeds the Pictl 97.5th % and s_micro_ and s_AMOC4 × 20_ over much of the tropical Indo-Pacific in a pattern that resembles the Southern Oscillation (black contours in Fig. 12g-i). While s_macro_ PR significantly exceeds the Pictl 97.5th % over the Arabian Sea, western tropical Pacific and along the South Pacific Convergence Zone, its significant exceedance relative to s_micro_ and s_AMOC4 × 20_ is limited to the Arabian Sea (black contours in Fig. 12j-l). Further analysis and experiments are needed to fully address the potential role of Krakatoa and Tarawera on ensemble spread and to understand the physical mechanisms governing a possible state-dependent response to these eruptions.

### Summary and implications

Leveraging the unique strategic design of the CESM2 Large Ensemble, we have quantified the impacts of initialization method (macro vs. micro perturbation) and ocean initial-condition memory on the evolution of ensemble spread in upper-ocean temperatures and surface climate variables over the first 6 decades of the historical simulations (1850–1909). Our results are based on decadal averages from which drift in the pre-industrial control simulation has been removed. To the best of our knowledge, this is the first time that these dual aspects of a Large Ensemble design have been quantified in a single state-of-the-art global coupled Earth system model.

Sensitivity of ensemble spread to the method of initialization is mainly limited to upper-ocean temperatures and SSTs over the SPNA and Arctic in the first two decades, with fractional differences of up to 30–90%. These differences are unlikely to have occurred by chance, as assessed by bootstrapped random sampling of the pre-industrial control simulation. An unexpected resurgence of statistically significant macro vs. micro ensemble spread differences occurs in the 4th decade (1880–1889) over the tropical Indo-Pacific in all variables examined, with fractional values ranging from 25 to 50%. We speculate that the large sequential volcanic eruptions of Krakatoa and Tarawera during this decade may have induced a state-dependent response that is preferentially expressed in the macro-initialized simulations, but it is also possible that these anomalies represent delayed initial condition memory associated with internal climate variability (see Zhang et al. 2023). Further work is needed to understand the origins and mechanisms underlying this unexpected signal.

There appear to be two stages of significant ocean initial-condition memory associated with the four chosen ocean initial states. The initial stage is focused in the North Atlantic and lasts for 3–4 decades in SST and 4–5 decades in upper-ocean temperatures. This stage can be explained by known AMOC dynamics. The second stage, located in the Indo-Pacific sector of the Southern Ocean, appears approximately 35-years after initialization and lasts for ~ 30–40 years in upper-ocean temperatures and SST. There is some evidence for eastward propagation of the signal across the Pacific sector of the Southern Ocean, with linkages to the subtropical southeast Pacific approximately 20 years later. Further work is needed to understand the dynamical mechanisms underlying the delayed appearance of significant ocean initial-condition memory in the Southern Ocean. We note that the ~ 20-year lag between the onset of ocean memory in the subtropics relative to higher latitudes of the southeast Pacific is quantitatively consistent with that found for the transient response to polar stratospheric ozone depletion (Dong et al. 2024). Additional investigation is also warranted to assess the predictability characteristics of each of the four AMOC ensembles individually, as they may differ depending on the phase and amplitude of the initial AMOC state.

Our results have implications for studies using the CESM2 Large Ensemble and for informing the experimental design of Large Ensembles in general. One major implication concerns the common practice of equating the ensemble-mean at a given time with the externally forced response. This only holds true once the memory of the initial conditions is lost. We have shown that in the CESM2 Large Ensemble, initial-condition memory in upper-ocean temperatures can last up to 4–5 decades depending on location, confounding the interpretation of the ensemble-mean as the externally forced response during the early decades of the historical simulations. To mitigate this issue, we recommend designing Large Ensembles with a buffer of several decades between the start date of the simulations and the start date of the intended period of analysis for ease of interpretation. For the same reason, we also recommend initializing Large Ensembles from a well spun-up Pictl simulation with minimal non-linear drift. With regard to initialization procedure, we recommend using a macro-initialization approach to guard against artificially limiting ensemble spread in the early decades of the historical simulations. We hope that our findings will stimulate future investigations of the physical mechanisms governing initial-condition memory effects and sensitivity of ensemble spread to methodological choices in the CESM2 Large Ensemble.

## Electronic Supplementary Material

Below is the link to the electronic supplementary material.

Supplementary Material 1

## Data Availability

Publicly available datasets were analyzed in this study. This data can be found here: https://www.cesm.ucar.edu/community-projects/lens2/data-sets .
