# Supplementary material for: Effects of macro vs. micro initialization and ocean initial-condition memory on the evolution of ensemble spread in the CESM2 large ensemble
Source: Clim Dyn. 2024 Dec 24;63(1):62. doi: 10.1007/s00382-024-07553-z (PMC11668834; doi:10.1007/s00382-024-07553-z)
Supplement: Supplementary file 1 — Supplementary Material 1 [file 382_2024_7553_MOESM1_ESM.pdf]

Supplemental Materials

Effects of macro *vs.* micro initialization and ocean initial-condition memory on the evolution of ensemble spread in the CESM2 Large Ensemble

Clara Deser[1*], Who M. Kim[1], Robert C. J. Wills[2], Isla R. Simpson[1], Steve Yeager[1], Gokhan Danabasoglu[1], Keith Rodgers[3] and Nan Rosenbloom[1]

[1] NSF National Center for Atmospheric Research, Boulder CO, USA
[2] Institute for Atmospheric and Climate Science, ETH Zürich, Switzerland
[3] WPI-Advanced Institute for Marine Ecosystem Change, Tohoku University, Japan

Submitted to *Climate Dynamics*

October 11, 2024

* Corresponding author: Dr. Clara Deser, Climate and Global Dynamics Laboratory, NCAR, 1850 Table Mesa Drive, Boulder CO 80305, cdeser@ucar.edu

This document contains 6 figures (Figs. S1-S6).

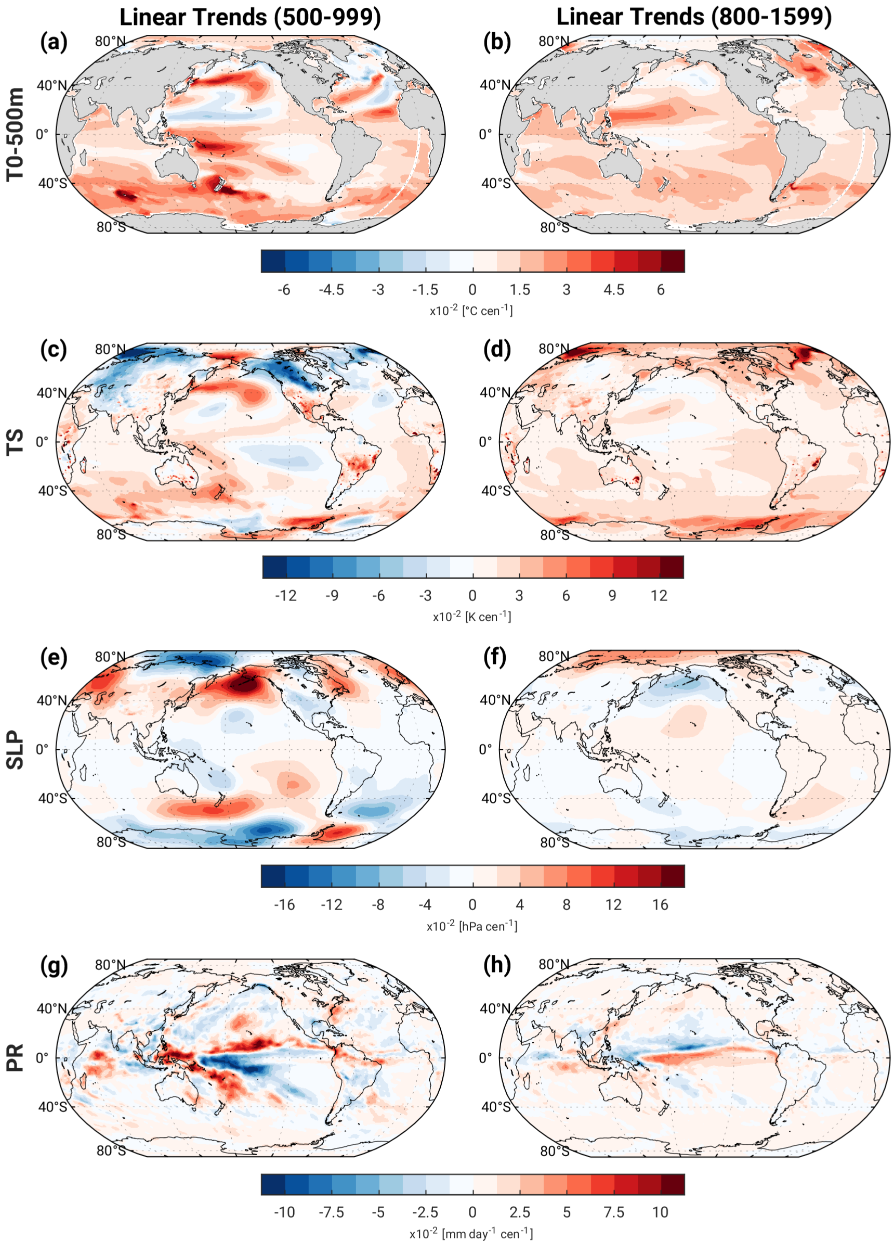

Fig. S1. Drift in the CESM2 pre-industrial control simulation during (left panels) years 500-999 and (right panels) years 800-1599 for (a,b) $T_{0\text{-}500m}$ (K century^{-1}) (c,d) TS (K century^{-1}), (e,f) SLP (hPa century^{-1})and (g,h) precipitation (mm day^{-1} century^{-1}).

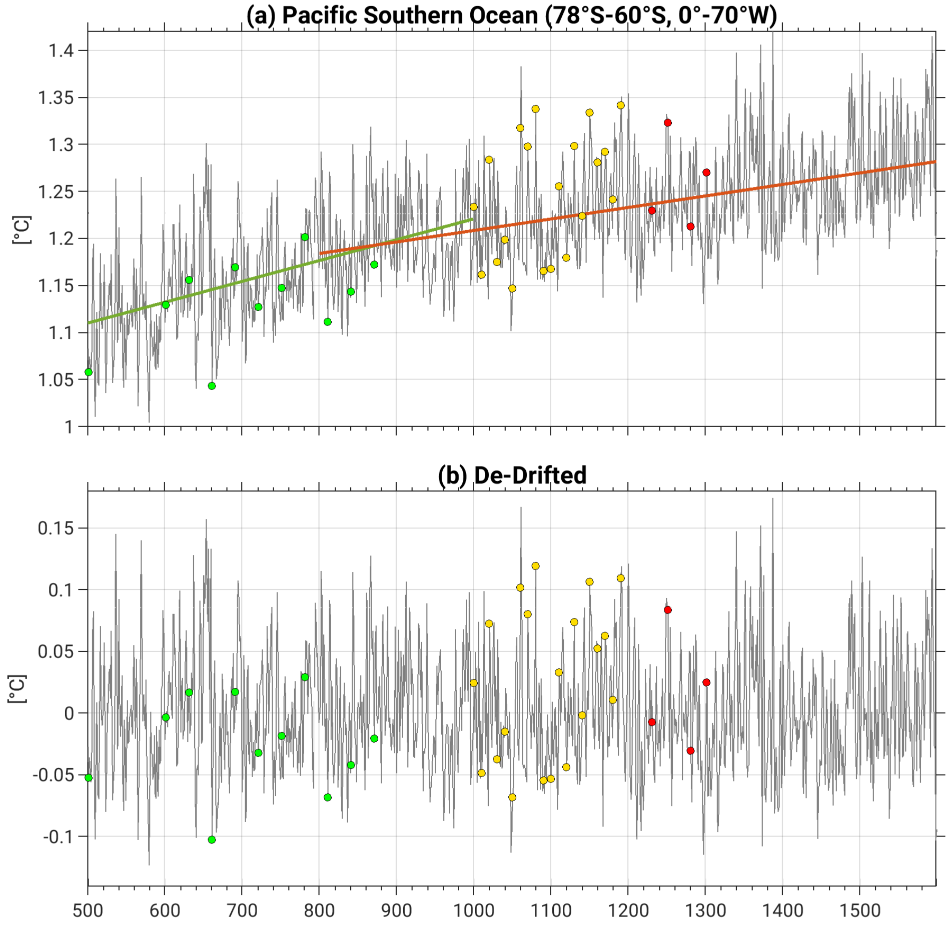

Fig. S2. Illustration of drift in the CESM2 pre-industrial control simulation. a) Annual mean upper ocean potential temperature ($T_{0\text{-}500m}$) timeseries averaged over the Pacific sector of the Southern Ocean (78°-60°S, 180W°-70°W) (top) before and (bottom) after de-drifting. In a), the solid green (red) line shows the best-fit linear trend over years 500-999 (800-1599). Years 800-899 (900-999) are de-drifted using the trend from years 500-999 (800-1599). The red circles mark the annual mean value in the first year of each member of AMOC1231, AMOC1251, AMOC1281 and AMOC1301. The yellow and green circles mark the annual mean value in the first year of each Macro20 and Macro11 simulation, respectively. See main text for details.

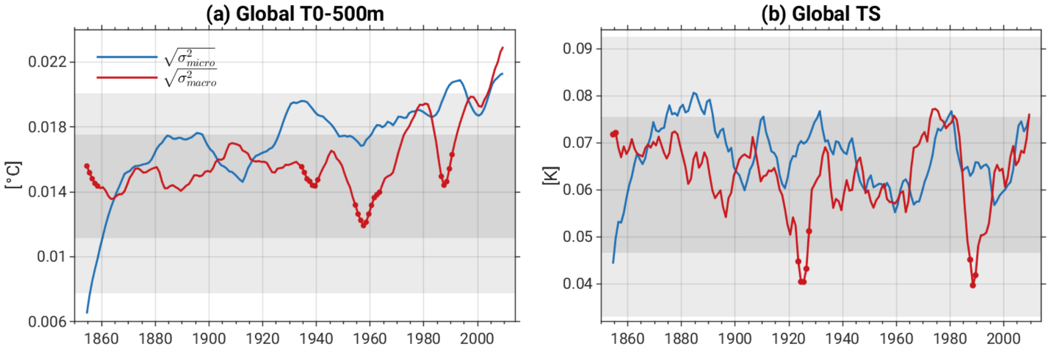

Fig. S3. Ensemble spread due to macro (red curves) and micro (blue curves) initialization for 10-year running means of global (a) $T_{0\text{-}500m}$ and (b) TS. Black circles on the red curves indicate that the spread due to macro-initialization is significantly different from that due to micro-initialization. Darker (lighter) gray shading denotes the 2.5th – 97.5th percentile (full) range based on the Pictl simulation.

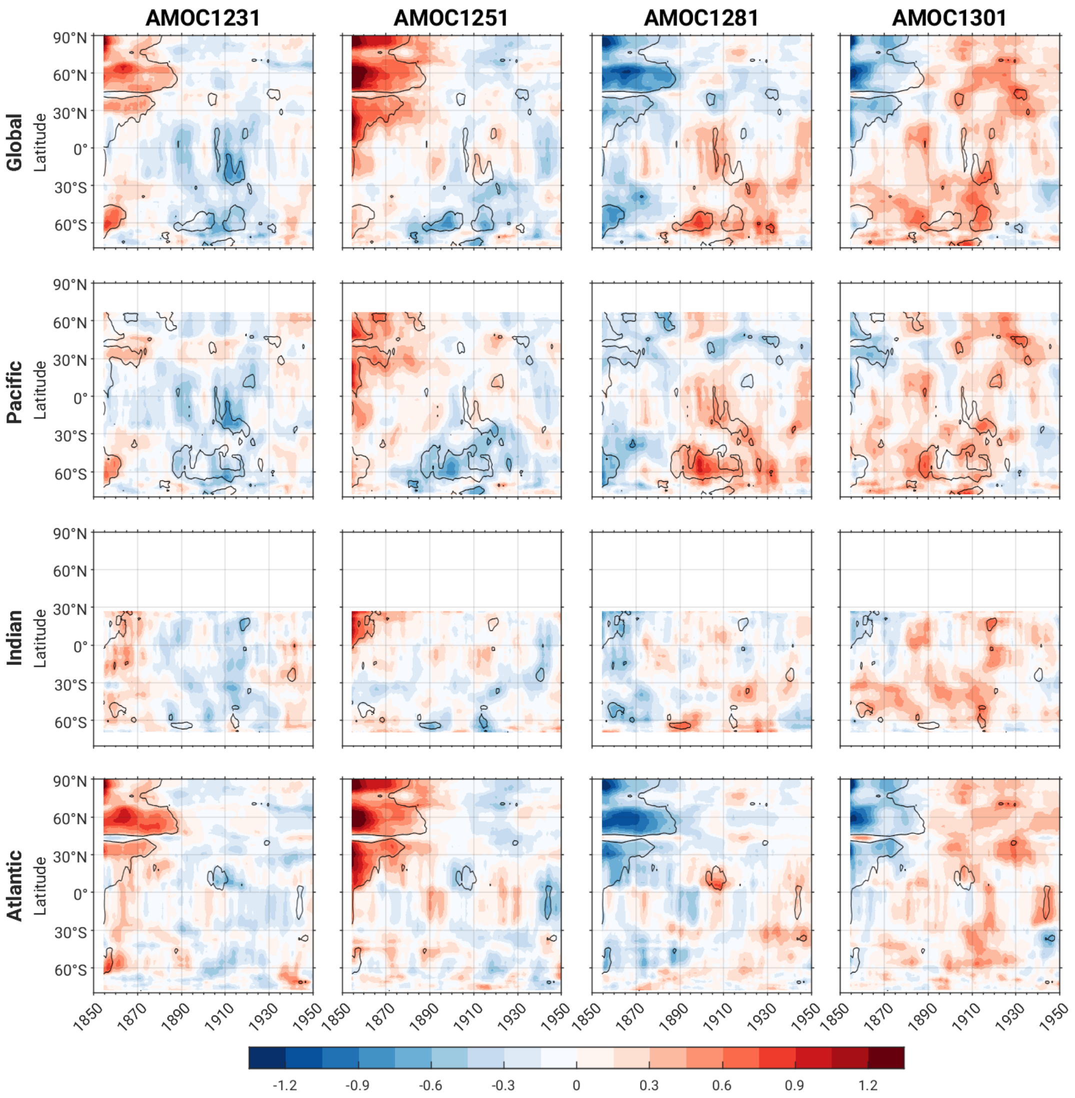

Fig. S4. Hovmöller diagrams of zonally averaged ensemble-mean normalized SST anomalies for each AMOC ensemble based on 10-year running means for the following domains: (top row) global; (2nd row) Pacific; (3rd row) Indian; and (bottom row) Atlantic. Anomalies are defined relative to the average of the 4 AMOC ensemble-means and normalized by the Pictl standard deviation. Contours of statistically significant χ_{ocean} are superimposed for context.

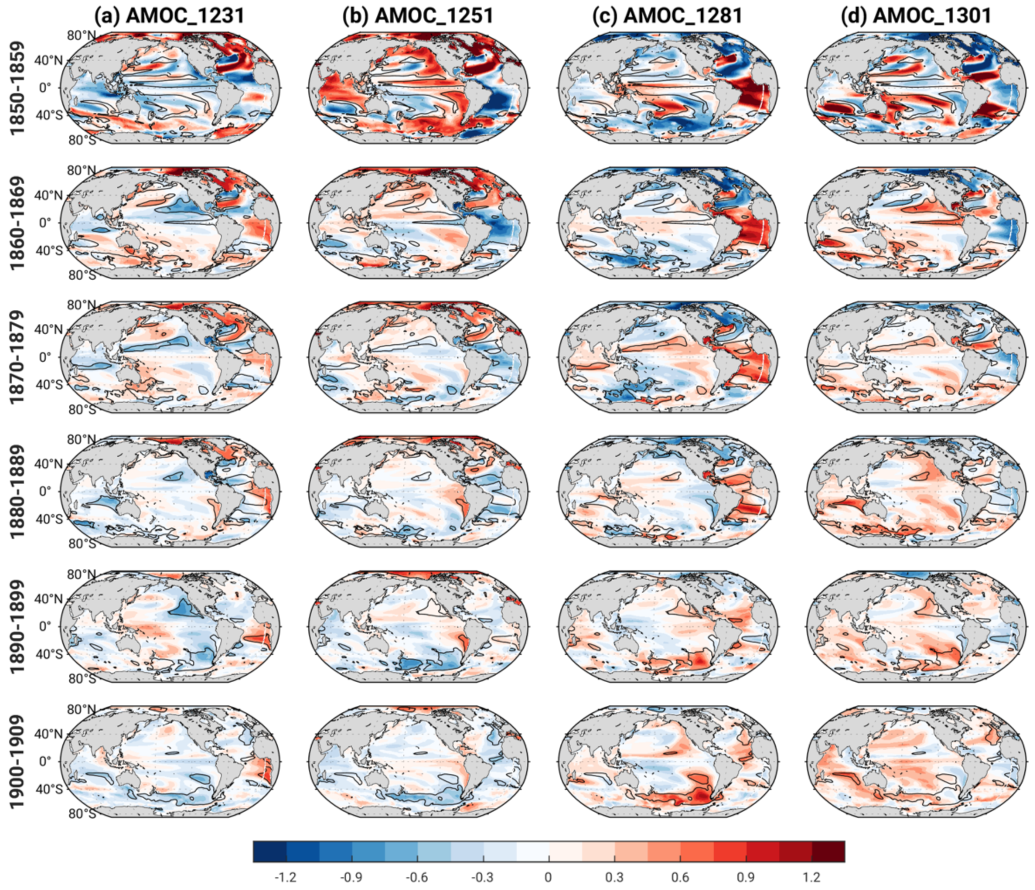

Fig. S5. Ensemble-mean normalized $T_{0\text{-}500m}$ anomalies over the first 6 decades of the historical simulations for each AMOC ensemble: (a) AMOC1231; (b) AMOC1251; (c) AMOC1281; and (d) AMOC1301. Anomalies are defined relative to the average of the 4 AMOC ensemble-means and normalized by the Pictl standard deviation. Contours of statistically significant χ_{ocean} are superimposed for context.

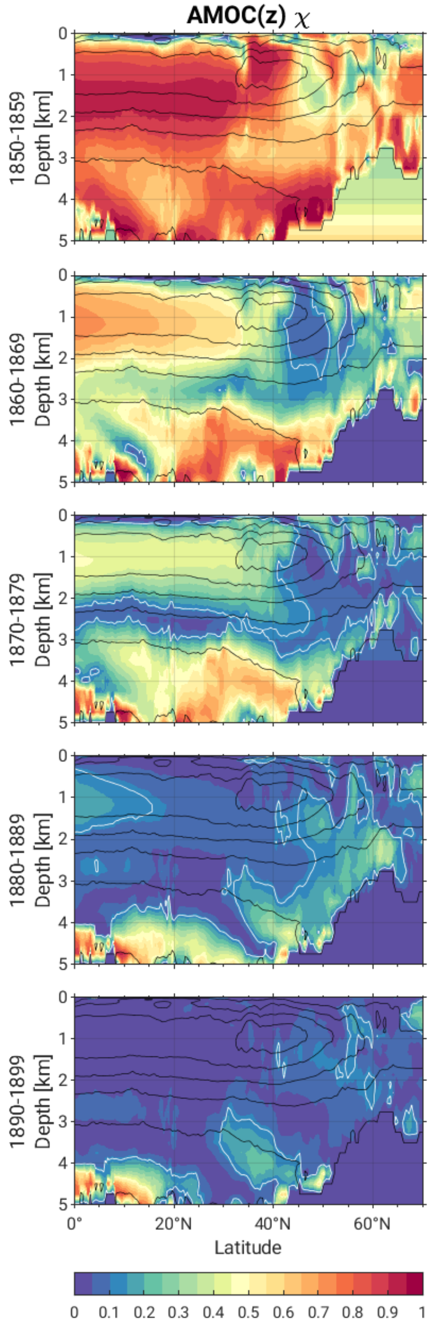

Fig. S6. Fraction of ensemble spread attributable to ocean initial-condition memory in the 4 AMOC ensembles (χ_{ocean}) based on decadal-means of AMOC in depth coordinates [AMOC(z)] over the first 5 decades of the historical simulations. White contours outline regions that are statistically significant at the 95% confidence level based on bootstrapping the Pictl simulation (see text for details).
